# Real-world use of multiplex point-of-care molecular testing or laboratory-based molecular testing for influenza-like illness in a 2021 to 2022 US outpatient sample

**DOI:** 10.1371/journal.pone.0313660

**Published:** 2024-11-11

**Authors:** Karen M. Stockl, Jamie Tucker, Anne Beaubrun, Julia M. Certa, Laura Becker, Jordan G. Chase

**Affiliations:** 1 Optum, Eden Prairie, Minnesota, United States of America; 2 Cepheid, Sunnyvale, California, United States of America; Carol Davila University of Medicine and Pharmacy, ROMANIA

## Abstract

While molecular testing is recommended for symptomatic patients suspected of having coronavirus disease 2019 (COVID-19), limited data are available examining real-world use of tests for severe acute respiratory syndrome coronavirus (SARS-CoV-2) and the impact of SARS-CoV-2 testing on patient outcomes. In this retrospective cohort study using de-identified administrative claims data in the Optum Labs Data Warehouse, we identified 2 groups of patients with ≥1 outpatient claims with a procedure code for SARS-CoV-2 testing between January 2021 and September 2022. Group 1 had ≥1 claims with CPT code 0240U or 0241U (“Xpert Xpress”) (N = 51,602); Group 2 had ≥1 claims for laboratory-based molecular testing (N = 317,192). Outcomes assessed on the identification date and through the 90-day follow-up included claims evidence of use of SARS-CoV-2, influenza, and respiratory syncytial virus (RSV) tests, diagnosis of active COVID-19, influenza, or RSV, and use of treatments (antivirals for COVID-19, influenza, and RSV and other treatments for COVID-19 and RSV). Patients in Group 1 had fewer tests for SARS-CoV-2, influenza, or RSV (mean ± standard deviation 1.6±1.4 versus 2.6±2.6, standardized difference -0.45), faster time to diagnosis of COVID-19 (median 0 versus 4 days, standardized difference -0.27) or influenza (median 0 versus 5 days, standardized difference -0.74), and faster time to treatment of COVID-19, influenza, or RSV (median 1 versus 5 days, standardized difference 0.16) than patients in Group 2. In this nationwide real-world study of outpatient testing, use of point-of-care molecular multiplex SARS-CoV-2 testing resulted in fewer claims for SARS-CoV-2, influenza, and RSV tests, faster time to diagnosis, and faster time to treatment than laboratory-based molecular testing.

## Introduction

Although different tests for detecting severe acute respiratory syndrome coronavirus 2 (SARS-CoV-2) have been developed since the start of the coronavirus disease 2019 (COVID-19) pandemic, limited data are available examining the use of these tests in clinical practice and the impact of these tests on patient outcomes. For symptomatic patients suspected of having COVID-19, the Infectious Diseases Society of America (IDSA) recommends use of molecular tests such as reverse transcriptase-polymerase chain reaction (RT-PCR) tests over rapid antigen tests for direct detection of SARS-CoV-2 in respiratory tract specimens [1]. Multiple test manufacturers and clinical laboratories have developed SARS-CoV-2 molecular tests that received emergency use authorization (EUA) under the COVID-19 public health emergency (PHE) initiated on February 4, 2020. Although the PHE expired on May 11, 2023, existing EUAs for devices relating to COVID-19 remain in effect under Section 564 of the Federal Food, Drug, and Cosmetic Act [2].

Molecular diagnostic tests for detection of SARS-CoV-2 have been authorized for use in different settings; some laboratory-based tests may be used only by trained personnel in laboratory facilities, while others have been authorized for use in point-of-care (POC) settings [3].

When evaluating patients presenting with symptoms of influenza-like illness (ILI), defined by the Centers for Disease Control and Prevention (CDC) as fever and cough and/or sore throat [4], clinicians may consider testing for influenza and/or respiratory syncytial virus (RSV) in addition to SARS-CoV-2 depending on the patient’s symptoms, risk factors, and viruses circulating in the community [5, 6]. Tests for influenza and RSV are available as antigen or molecular tests and as single agent or multiplex tests. The IDSA recommends rapid molecular assays over rapid influenza diagnostic tests to improve detection of influenza virus in the outpatient setting [7]. Some rapid molecular tests for influenza can be administered in the POC setting [8].

Xpert^®^ Xpress CoV-2/Flu/RSV plus and Xpert^®^ Xpress SARS-CoV-2/Flu/RSV (collectively, “Xpert Xpress”; Cepheid, Sunnyvale, CA) are rapid multiplex RT-PCR tests for respiratory viruses that can be used at the point of care. Xpert Xpress provides results for SARS-CoV-2, influenza A, and influenza B (3-plex test) or SARS-CoV-2, influenza A, influenza B, and RSV (4-plex test) [9–11]. Xpert Xpress SARS-CoV-2/Flu/RSV received EUA in September 2020 and Xpert Xpress CoV-2/Flu/RSV plus added a third gene target for SARS-CoV-2 detection and received EUA in September 2021.

The aim of this study was to compare the real-world patient demographics and clinical characteristics, use of testing for respiratory viruses, and use of antiviral or immunomodulator treatments between patients presenting with symptoms of ILI in the outpatient setting who received testing with Xpert Xpress and those who received laboratory-based molecular testing for SARS-CoV-2 with or without influenza or RSV.

## Methods

### Design and data source

This was a descriptive, retrospective cohort study using de-identified administrative claims data from July 1, 2020 through September 30, 2022 (study period) for commercially insured and Medicare Advantage enrollees in the Optum Labs Data Warehouse (OLDW). The OLDW contains longitudinal medical and pharmacy claims and enrollment records for enrollees and patients, representing a mixture of ages and geographical regions across the United States.

### Ethics statement

This study utilized de-identified data using the “Expert Determination” de-identification method in compliance with 45 C.F.R. § 164.514(b)(1), and as a result did not involve human subjects nor was personal health information collected. De-identified information is exempt from Institutional Review Board review. The authors had no access to information that could individually identify participants during or after data collection.

### Identification of study patients

**Fig 1:**
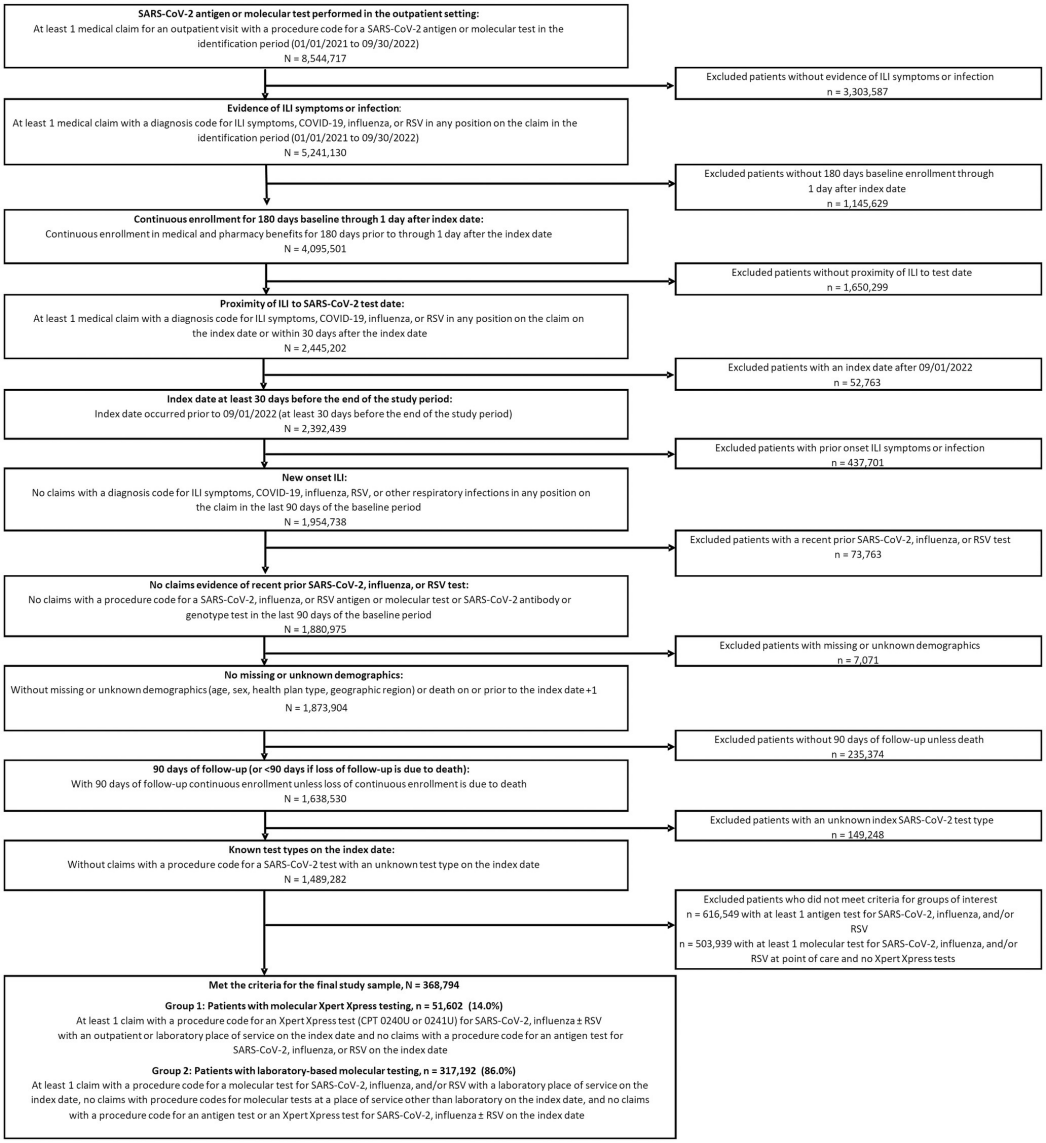
Study patient identification and attrition flowchart.

As shown in Fig 1, patients were initially identified if they had ≥1 medical claims for an outpatient visit with a procedure code for testing for SARS-CoV-2 (S1 Table) and ≥1 medical claims with a diagnosis code for ILI symptoms or infection (S2 Table) in the identification period (January 1, 2021 to September 30, 2022). The date of the patient’s first claim for a SARS-CoV-2 test in the outpatient setting was assigned as the index date. Patients were required to have continuous enrollment in the 180-day baseline period prior to the index date, and in the 90-day follow-up period after the index date (or <90-day follow-up period if loss of continuous enrollment was due to death).

The following 2 study groups of interest were selected: Group 1 (Xpert Xpress testing) had ≥1 claims with CPT code 0240U or 0241U with an outpatient visit or laboratory place of service on the index date; Group 2 (laboratory-based molecular testing) had ≥1 claims with procedure code for a molecular test for SARS-CoV-2, influenza, and/or RSV (S3 Table) with a laboratory place of service on the index date.

### Study measures

Patient characteristics were assessed during the 180-day baseline period. Baseline Charlson comorbidity index was calculated using methods defined by Quan et al. [12] Baseline high-risk conditions were selected for evaluation based on the risk factors for influenza complications outlined by the CDC [13]. The full list of respiratory and non-respiratory risk factors is included in S4 and S5 Tables, respectively. Patients were identified with a high-risk condition if they had ≥2 medical claims ≥30 days apart with a diagnosis or procedure code for the risk factor or ≥1 claims for a systemic corticosteroid or immunosuppressive medication during the baseline period. Prior COVID-19 and influenza vaccinations were identified using claims with a National Drug Code (NDC) or procedure code for the vaccine in the baseline period.

Study outcomes including number of tests, evidence of and time to first active (non-historical) diagnosis, use of antiviral or immunomodulator treatments, and time to first treatment were assessed during the 90-day follow-up period (or <90 days in cases of death) which included the index date.

On the index date and in the follow-up period, tests that the patient received for SARS-CoV-2, influenza, and RSV were identified from medical claims with any place of service (outpatient or inpatient) with a procedure code for SARS-CoV-2, influenza, or RSV antigen or molecular tests (S3 Table).

Evidence of diagnosis of active infection with COVID-19, influenza, and/or RSV was defined as having ≥1 medical claims with a non-laboratory place of service with a diagnosis code for active COVID-19, influenza, or RSV (S6 Table) in any position on the claim during the follow-up period (inclusive of the index date). Time to first evidence of diagnosis of active infection with COVID-19, influenza, or RSV was measured among patients who met the criteria for evidence of diagnosis of a condition of interest and reported as the days from the index date to the first non-laboratory visit claim with a diagnosis code for the condition of interest (COVID-19, influenza, or RSV) in the follow-up period.

Co-infection was defined as having ≥2 active conditions of interest (COVID-19, influenza, and/or RSV) in the follow-up period and the diagnosed conditions of interest occurred within the same 14-day window as the first identified diagnosed condition of interest. Time from index date to first co-infection was measured as the time from the index date to the first non-laboratory medical claim with a diagnosis code for the second co-infected condition of interest in the follow-up period.

Treatments for COVID-19, influenza, and RSV were identified with ≥1 claims for a treatment (specific treatments listed in S7 Table) in the follow-up period. For COVID-19, treatments included antivirals, immunomodulators and Janus kinase (JAK) inhibitors, monoclonal antibodies, hydroxychloroquine, and convalescent plasma (CCP). For influenza, treatments included antivirals. For RSV, treatments included antivirals, monoclonal antibodies, and immune globulins. Use of a treatment and time to first treatment (number of days from index date to the first claim for a treatment) were measured among all patients in each group and among the subset of patients with a diagnosis code for active COVID-19, influenza, or RSV.

### Statistical analyses

Analyses were conducted using SAS version 9.4 (SAS Institute, Cary, NC). Study measures were evaluated using descriptive summary statistics. Standardized differences were used to compare Group 1 with Group 2 instead of Chi-square tests and t-tests which would have produced p-values that were difficult to interpret due to the large sample sizes of patients identified in both groups of this study [14–16]. Standardized difference values >0.10 or <-0.10 were considered to have significant imbalance between proportions or means [15, 16].

## Results

Overall, 368,794 patients met the inclusion and exclusion criteria, 51,602 (14.0%) in Group 1 and 317,192 (86.0%) in Group 2 (Fig 1).

### Patient characteristics

Patient characteristics are shown in Table 1. Group 1 was older, on average, than Group 2 (mean 49.8 years versus 42.2 years, standardized difference 0.30). Age distribution was bimodal with a higher proportion of Group 1 with age <5 years (8.3% versus 4.9%, standardized difference 0.14) and with age ≥65 years (41.5% versus 22.8%, standardized difference 0.41) than Group 2. Group 1 was more likely to have Medicare Advantage insurance (47.0% versus 23.9%, standardized difference 0.50) and to reside in the Midwest (29.0% versus 21.6%, standardized difference 0.17) than Group 2.

**Table 1:** Patient characteristics.

	Group 1 Xpert Xpress N = 51,602	Group 2 Laboratory-based Molecular N = 317,192	Std. Diff.*
**Age based on year of index date**			
Mean years (SD), median	49.8 (27.4), 57	42.2 (23.9), 42	0.30
0–4 years	4275 (8.3%)	15,568 (4.9%)	0.14
5–11 years	3289 (6.4%)	27,918 (8.8%)	-0.09
12–17 years	2430 (4.7%)	22,002 (6.9%)	-0.10
18–44 years	10,003 (19.4%)	101,878 (32.1%)	-0.29
45–64 years	10,169 (19.7%)	77,568 (24.5%)	-0.11
65+ years	21,436 (41.5%)	72,258 (22.8%)	0.41
**Sex**			
Female	27,767 (53.8%)	171,884 (54.2%)	-0.01
Male	23,835 (46.2%)	145,308 (45.8%)	0.01
**Health insurance type**			
Commercial	27,338 (53.0%)	241,247 (76.1%)	-0.50
Medicare Advantage	24,264 (47.0%)	75,945 (23.9%)	0.50
**Geographic region**			
Northeast	5430 (10.5%)	39,971 (12.6%)	-0.07
Midwest	14,941 (29.0%)	68,352 (21.6%)	0.17
South	21,891 (42.4%)	141,877 (44.7%)	-0.05
West	9340 (18.1%)	66,992 (21.1%)	-0.08
**Charlson Comorbidity Index in the baseline period**			
Mean (SD), median	0.70 (1.38), 0	0.35 (0.97), 0	0.29
0	35,953 (69.7%)	262,712 (82.8%)	-0.31
1 to 2	10,677 (20.7%)	41,732 (13.2%)	0.20
3 to 4	3539 (6.9%)	9299 (2.9%)	0.18
5+	1433 (2.8%)	3449 (1.1%)	0.12
**Risk factors for ILI complications in the baseline period**			
Any respiratory risk factor	3143 (6.1%)	7550 (2.4%)	0.19
Asthma	720 (1.4%)	2940 (0.9%)	0.04
Chronic lung disease	2572 (5.0%)	4934 (1.6%)	0.19
Any other risk factor	22,544 (43.7%)	100,582 (31.7%)	0.25
Diabetes	6320 (12.3%)	20,792 (6.6%)	0.20
Neurologic conditions	6383 (12.4%)	24,671 (7.8%)	0.15
Immunocompromised status	264 (0.5%)	1010 (0.3%)	0.03
Transplant	127 (0.3%)	419 (0.1%)	0.03
Immunosuppressive medications	9369 (18.2%)	41,403 (13.1%)	0.14
Cancer	1536 (3.0%)	5185 (1.6%)	0.09
Tuberculosis	<11 (0.0%)	23 (0.0%)	0
Kidney disease	3207 (6.2%)	9072 (2.9%)	0.16
Liver disease	439 (0.9%)	1953 (0.6%)	0.03
Heart disease	6045 (11.7%)	17,583 (5.5%)	0.22
Congenital heart defects	59 (0.1%)	204 (0.1%)	0.02
Stroke	400 (0.8%)	1018 (0.3%)	0.06
Metabolic disorders	6503 (12.6%)	25,502 (8.0%)	0.15
Current or history of smoking	1241 (2.4%)	3019 (1.0%)	0.11
Mood disorders	2909 (5.6%)	15,317 (4.8%)	0.04
Blood disorders	2065 (4.0%)	6970 (2.2%)	0.10
Pregnancy	448 (0.9%)	2933 (0.9%)	-0.01
Obesity	1959 (3.8%)	8962 (2.8%)	0.05
**Claims evidence of vaccination in the baseline period**			
SARS-CoV-2 vaccination	6332 (12.3%)	51,000 (16.1%)	-0.11
Influenza vaccination	13,497 (26.2%)	75,243 (23.7%)	0.06

Values are shown as number (%) of patients unless noted otherwise.

ILI = influenza-like illness, SARS-CoV-2 = severe acute respiratory syndrome coronavirus 2, SD = standard deviation, Std. Diff. = standardized difference

*Represents the standardized difference for the comparison of patients with Xpert Xpress versus laboratory-based molecular tests. A standardized difference of >0.10 or <-0.10 was considered a significant imbalance between proportions or means.

Group 1 had a higher baseline comorbidity burden (mean Charlson index 0.70 versus 0.35, standardized difference 0.29) and were more likely to have a respiratory risk factor (6.1% versus 2.4%, standardized difference 0.19) or other risk factor (43.7% versus 31.7%, standardized difference 0.25) than patients in Group 2. Claims evidence of SARS-CoV-2 vaccination was lower in Group 1 than Group 2 (12.3% versus 16.1% of patients, standardized difference -0.11).

**Fig 2:**
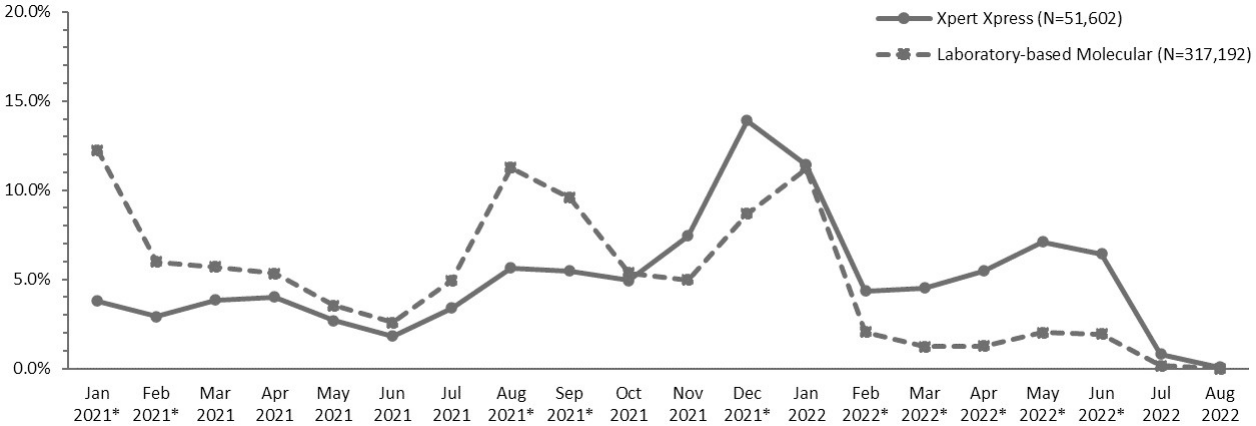
Distribution of patients in Group 1 (Xpert Xpress) and Group 2 (Laboratory-based molecular testing) by month of index test. *Denotes a significant difference (absolute value of standardized difference >0.1) between patients with Xpert Xpress versus laboratory-based molecular testing.

Fig 2 illustrates the distribution of patients in Group 1 and Group 2 by month of their index test. Patients in Group 1 were more likely to have their index test in December 2021 or February through June 2022 than patients in Group 2.

On the index date, 302,794 (95.5%) patients in Group 2 were tested for SARS-CoV-2 only, while 8,230 (2.6%) were tested for SARS-CoV-2 and influenza, 6,086 (1.9%) were tested for SARS-CoV-2, influenza, and RSV, and 82 (<0.1%) were tested for SARS-CoV-2 and RSV. In comparison, Group 1 had higher percentages of patients with testing for SARS-CoV-2 and influenza (11.4%, n = 5,892, standardized difference 0.35) and with testing for SARS-CoV-2, influenza, and RSV (88.6%, n = 45,710, standardized difference 3.54) on the index date.

The site of testing on the index date was POC only for most (97.0%, n = 50,050) of the patients in Group 1, while 1,292 (2.5%) were tested in the laboratory only and 260 (0.5%) were tested both at POC and in the laboratory setting. POC testing occurred in the emergency department (ED) for 16,024 (31.1%) of patients in Group 1. The site of testing on the index date was laboratory only for all the patients in Group 2 as this was required for inclusion in Group 2.

### Number of tests administered

Patients in Group 1 had fewer claims for tests for SARS-CoV-2, influenza, or RSV on the index date (mean 1.2 versus 1.6 claims, standardized difference -0.54), after the index date through the end of the follow-up period (mean 0.4 versus 1.0 claims, standardized difference -0.29), and in the entire follow-up period including the index date (mean 1.6 versus 2.6 claims, standardized difference -0.45) than patients in Group 2 (Table 2). As most patients in Group 2 did not have a test for influenza or RSV, these results were primarily driven by tests for SARS-CoV-2 with Group 1 having fewer claims for SARS-CoV-2 tests (mean 1.6 versus 2.5 claims, standardized difference -0.45), but more claims for influenza (mean 1.3 versus 0.1 claims, standardized difference 1.81), and RSV tests (mean 1.1 versus 0.1, standardized difference 1.79) than Group 2 in the entire follow-up period including the index date. Among patients with ≥1 additional test after the index date, the time from index date to first additional test was longer for Group 1 than Group 2 (median 26 versus 13 days, standardized difference 0.38).

**Table 2:** Tests on the index date, after the index date to the end of the 90-day follow-up, and on the index date to the end of the 90-day follow-up.

	Group 1 Xpert Xpress N = 51,602	Group 2 Laboratory-based Molecular N = 317,192	Std. Diff.*	Group 1 Xpert Xpress N = 51,602	Group 2 Laboratory-based Molecular N = 317,192	Std. Diff.*	Group 1 Xpert Xpress N = 51,602	Group 2 Laboratory-based Molecular N = 317,192	Std. Diff.*
**Claims for SARS-CoV-2, influenza, or RSV tests**	**On index date**			**After index date to end of follow-up**			**On the index date to end of follow-up**		
Number of claims, mean (SD), median	1.2 (0.6), 1	1.6 (0.8), 2	-0.54	0.4 (1.2), 0	1.0 (2.4), 0	-0.29	1.6 (1.4), 1	2.6 (2.6), 2	-0.45
Patients with no claims, n (%)	-	-	-	40,875 (79.2%)	207,438 (65.4%)	0.31	-	-	-
Patients with 1 claim, n (%)	43,997 (85.3%)	157,651 (49.7%)	0.82	5332 (10.3%)	37,129 (11.7%)	-0.04	34,949 (67.7%)	105,037 (33.1%)	0.74
Patients with 2 claims, n (%)	5542 (10.7%)	145,481 (45.9%)	-0.85	3088 (6.0%)	36,531 (11.5%)	-0.20	9063 (17.6%)	117,430 (37.0%)	-0.45
Patients with 3 claims, n (%)	1360 (2.6%)	4381 (1.4%)	0.09	1034 (2.0%)	11,269 (3.6%)	-0.09	4027 (7.8%)	29,538 (9.3%)	-0.05
Patients with 4+ claims, n (%)	703 (1.4%)	9679 (3.1%)	-0.12	1273 (2.5%)	24,825 (7.8%)	-0.24	3563 (6.9%)	65,187 (20.6%)	-0.40
Days from index date to the first additional test for patients with ≥1 claims for a test, mean (SD), median	-	-	-	32.6 (26.1), 26	23.2 (23.8), 13	0.38	-	-	-
**Claims for SARS-CoV-2 tests**	**On index date**			**After index date to end of follow-up**			**On the index date to end of follow-up**		
Number of claims, mean (SD), median	1.2 (0.6), 1	1.6 (0.8), 1	-0.55	0.4 (1.2), 0	0.9 (2.3), 0	-0.29	1.6 (1.3), 1	2.5, (2.5), 2	-0.45
Patients with no claims, n (%)	-	-	-	41,084 (79.6%)	209,182 (66.0%)	0.31	-	-	-
Patients with 1 claim, n (%)	44,115 (85.5%)	159,072 (50.2%)	0.82	5538 (10.7%)	39,130 (12.3%)	-0.05	35,213 (68.2%)	107,043 (33.8%)	0.74
Patients with 2 claims, n (%)	5495 (10.7%)	146,965 (46.3%)	-0.86	3060 (5.9%)	36,589 (11.5%)	-0.20	9243 (17.9%)	120,717 (38.1%)	-0.46
Patients with 3 claims, n (%)	1345 (2.6%)	2515 (0.8%)	0.14	847 (1.6%)	9738 (3.1%)	-0.09	4027 (7.8%)	28,782 (9.1%)	-0.05
Patients with 4+ claims, n (%)	647 (1.3%)	8640 (2.7%)	-0.11	1073 (2.1%)	22,553 (7.1%)	-0.24	3119 (6.0%)	60,650 (19.1%)	-0.40
Days from index date to the first additional test for patients with ≥1 claims for a test, mean (SD), median	-	-	-	32.7 (26.1), 26	23.3 (23.8), 13	0.38	-	-	-
**Claims for influenza tests**	**On index date**			**After index date to end of follow-up**			**On the index date to end of follow-up**		
Number of claims, mean (SD), median	1.2 (0.5), 1	0.1 (0.3), 0	2.58	0.1 (0.5), 0	0.1 (0.4), 0	0.12	1.3 (0.8), 1	0.1 (0.5), 0	1.81
Patients with no claims, n (%)	-	302,751 (95.5%)	-6.48	46,593 (90.3%)	300,707 (94.8%)	-0.17	-	287,761 (90.7%)	-4.42
Patients with 1 claim, n (%)	44,763 (86.8%)	12,153 (3.8%)	3.01	3626 (7.0%)	10,137 (3.2%)	0.17	40,445 (78.4%)	20,101 (6.3%)	2.13
Patients with 2 claims, n (%)	5242 (10.2%)	1603 (0.5%)	0.44	1019 (2.0%)	4908 (1.6%)	0.03	7985 (15.5%)	6871 (2.2%)	0.48
Patients with 3 claims, n (%)	1086 (2.1%)	477 (0.2%)	0.19	216 (0.4%)	700 (0.2%)	0.04	2063 (4.0%)	1334 (0.4%)	0.25
Patients with 4+ claims, n (%)	511 (1.0%)	208 (0.1%)	0.13	148 (0.3%)	740 (0.2%)	0.01	1109 (2.2%)	1125 (0.4%)	0.16
Days from index date to the first additional test for patients with ≥1 claims for a test, mean (SD), median	-	-	-	33.4 (26.9), 27	26.1 (26.1), 16	0.27	-	-	-
**Claims for RSV tests**	**On index date**			**After index date to end of follow-up**			**On the index date to end of follow-up**		
Number of claims, mean (SD), median	1.0 (0.6), 1	0.0 (0.2), 0	2.17	0.1 (0.4), 0	0.0 (0.3), 0	0.21	1.1 (0.8), 1	0.1 (0.4), 0	1.79
Patients with no claims, n (%)	5886 (11.4%)	310,896 (98.0%)	-3.53	47,742 (92.5%)	311,306 (98.1%)	-0.27	5771 (11.2%)	305,618 (96.4%)	-3.28
Patients with 1 claim, n (%)	39,615 (76.8%)	4608 (1.5%)	2.43	2979 (5.8%)	4370 (1.4%)	0.24	36,449 (70.6%)	8187 (2.6%)	2.00
Patients with 2 claims, n (%)	4689 (9.1%)	1130 (0.4%)	0.42	632 (1.2%)	966 (0.3%)	0.11	6989 (13.5%)	2164 (0.7%)	0.52
Patients with 3 claims, n (%)	970 (1.9%)	406 (0.1%)	0.18	152 (0.3%)	292 (0.1%)	0.05	1546 (3.0%)	715 (0.2%)	0.22
Patients with 4+ claims, n (%)	442 (0.9%)	152 (0.1%)	0.12	97 (0.2%)	258 (0.1%)	0.03	847 (1.6%)	508 (0.2%)	0.16
Days from index date to the first additional test for patients with ≥1 claims for a test, mean (SD), median	-	-	-	32.9 (26.9), 26	27.6 (26.0), 18	0.20	-	-	-

ILI = influenza-like illness, SARS-CoV-2 = severe acute respiratory syndrome coronavirus 2, RSV = respiratory syncytial virus, SD = standard deviation, Std. Diff. = standardized difference

*Represents the standardized difference for the comparison of patients with Xpert Xpress versus laboratory-based molecular tests. A standardized difference of >0.10 or <-0.10 was considered a significant imbalance between proportions or means.

### Evidence of and time to diagnosis of active infection

Compared with Group 2, Group 1 had a higher proportion of patients with a diagnosis code for active COVID-19 (28.0% versus 20.6%, standardized difference 0.17), for active influenza (3.6% versus 0.6%, standardized difference 0.21), and for active RSV (2.4% versus 0.2%, standardized difference 0.20) in the follow-up period (Table 3).

**Table 3:** Use of treatments and diagnosis of COVID-19, influenza, and RSV the 90-day follow-up period including the index date.

	Group 1 Xpert Xpress N = 51,602	Group 2 Laboratory-based Molecular N = 317,192	Std. Diff.*
**Use of treatments for COVID-19, influenza, or RSV in the 90-day follow-up period including the index date**			
**COVID-19, influenza, or RSV treatments**			
≥1 claims for a COVID-19, influenza, or RSV treatment^1^^–^^6^	3842 (7.4%)	13,544 (4.3%)	0.14
Days from index date to the first treatment for COVID-19, influenza, or RSV among patients with ≥1 claims for a treatment for COVID-19, influenza, or RSV, mean (SD), median	9.7 (19.5), 1	12.7 (19.2), 5	-0.16
**COVID-19 treatments**			
≥1 claims for a COVID-19 treatment^1^	3002 (5.8%)	12,258 (3.9%)	0.09
≥1 claims for a COVID-19 antiviral^2^	1514 (2.9%)	5182 (1.6%)	0.09
≥1 claims for a COVID-19 immunomodulator or JAK inhibitor^3^	188 (0.4%)	823 (0.3%)	0.02
≥1 claims for a COVID-19 monoclonal antibody^4^	1093 (2.1%)	5061 (1.6%)	0.04
≥1 claims for hydroxychloroquine	369 (0.7%)	1939 (0.6%)	0.01
≥1 claims for COVID-19 convalescent plasma (CCP)	75 (0.2%)	524 (0.2%)	-0.01
Days from index date to the first treatment for COVID-19 among patients with ≥1 claims for a treatment for COVID-19, mean (SD), median	10.6 (19.9), 2	11.8 (17.8), 5	-0.06
**Influenza treatments**			
≥1 claims for an influenza antiviral treatment^5^	823 (1.6%)	1247 (0.4%)	0.12
Days from index date to the first treatment for influenza among patients with ≥1 claims for a treatment for influenza, mean (SD), median	6.1 (17.6), 0	21.1 (28.5), 3	-0.63
**RSV treatments**			
≥1 claims for an RSV treatment^6^	55 (0.1%)	144 (0.1%)	0.02
Days from index date to the first treatment for RSV among patients with ≥1 claims for a treatment for RSV, mean (SD), median	29.4 (23.4), 21	23.6 (23.2), 16	0.25
**Diagnosis of COVID-19 and use of COVID-19 treatments in the 90-day follow-up period including the index date**			
≥1 non-laboratory medical claims with a diagnosis code for active COVID-19	14,433 (28.0%)	65,224 (20.6%)	0.17
Days from index date to first non-laboratory medical claim with a diagnosis code for active COVID-19 among patients with a diagnosis code for active COVID-19, mean (SD), median	4.9 (15.2), 0	8.9 (14.2), 4	-0.27
≥1 non-laboratory medical claims with a diagnosis code for active COVID-19 with use of ≥1 COVID-19 treatments^1^, n (% of patients with diagnosis of active COVID-19)	2634 (18.2%)	10,397 (15.9%)	0.06
Days from index date to the first treatment for COVID-19 among patients with ≥1 claims for a COVID-19 treatment and a diagnosis code for active COVID-19, mean (SD), median	7.9 (17.1), 1	9.2 (14.2), 5	-0.08
**Diagnosis of influenza and use of influenza treatments in the 90-day follow-up period including the index date**			
≥1 non-laboratory medical claims with a diagnosis code for active influenza	1857 (3.6%)	1792 (0.6%)	0.21
Days from index date to first non-laboratory medical claim with a diagnosis code for active influenza among patients with a diagnosis code for active influenza, mean (SD), median	4.1 (14.9), 0	20.3 (27.0), 5	-0.74
≥1 non-laboratory medical claims with a diagnosis code for active influenza with use of ≥1 influenza treatments^5^, n (% of patients with a diagnosis code for active influenza)	606 (32.6%)	554 (30.9%)	0.04
Days from index date to the first treatment for influenza among patients with ≥1 claims for a treatment and a diagnosis code for active influenza, mean (SD), median	4.7 (15.3), 0	26.1 (29.8), 14	-0.90
**Diagnosis of RSV and use of RSV treatments in the 90-day follow-up period including the index date**			
≥1 non-laboratory medical claims with a diagnosis for active RSV	1224 (2.4%)	462 (0.2%)	0.20
Days from index date to first non-laboratory medical claim with a diagnosis code for active RSV among patients with a diagnosis code for active RSV, mean (SD), median	4.7 (14.8), 0	26.7 (28.6), 14	-0.97
≥1 non-laboratory medical claims with a diagnosis for active RSV with use of ≥1 RSV treatments^6^, n (% of patients with a diagnosis code for active RSV)	<11 (<0.9%)	0	-
**Diagnosis of 2 or more conditions of interest and co-infection in the 90-day follow-up period including the index date**			
≥2 active conditions of interest (COVID-19, influenza, RSV)	331 (0.6%)	360 (0.1%)	0.09
Co-infection^7^ with ≥2 conditions of interest (COVID-19, influenza, RSV)	214 (0.4%)	234 (0.1%)	0.07
Days from index date to first diagnosis of co-infection^8^ among patients with a co-infection, mean (SD), median	2.4 (10.1), 0	11.6 (15.3), 6	-0.72

Values are shown as number (%) of patients unless noted otherwise.

COVID-19 = coronavirus disease 2019, JAK = Janus kinase, RSV = respiratory syncytial virus Std. Diff. = standardized difference

^1^. COVID-19 treatments included COVID-19 antivirals, COVID-19 immunomodulators and JAK inhibitors, COVID-19 monoclonal antibodies, hydroxychloroquine, and COVID-19 convalescent plasma (CCP). See footnotes 2–4 below.

^2^. COVID-19 antivirals included ritonavir-boosted nirmatrelvir, remdesivir, and molnupiravir.

^3^. COVID-19 immunomodulators and JAK inhibitors included baricitinib, tofacitinib, tocalizumab, and sarilumab.

^4^. COVID-19 monoclonal antibodies included bamlanivimab, etesevimab, casirivimab, imdevimab, sotrovimab, bebtelovimab, tixagevimab, and cilgavimab.

^5^. Influenza treatments/antivirals included baloxavir, oseltamivir, peramivir, rimatadine, and zanamivir.

^6^. RSV treatments included RSV antivirals (ribavirin), RSV monoclonal antibodies (palivizumab), and immune globulins.

^7^. Patients were identified with a co-infection if they met the criteria for having evidence of ≥2 active conditions of interest (COVID-19, influenza, and/or RSV) in the 90-day follow-up period and the diagnosed conditions of interest occurred within the same 14-day window (14-day co-infection window) as the first identified diagnosed condition of interest.

^8^. Time from index date to first co-infection was measured as the time from the index date to the first non-laboratory medical claim with a diagnosis code for the second co-infected condition of interest in the 90-day follow-up period.

*Represents the standardized difference for the comparison of patients with Xpert Xpress versus laboratory-based molecular tests. A standardized difference of >0.10 or <-0.10 was considered a significant imbalance between proportions or means.

Among patients with a diagnosis code for the condition of interest, patients in Group 1 had a faster time from index date to the first diagnosis code for active COVID-19 (median 0 versus 4 days, standardized difference -0.27), for active influenza (median 0 versus 5 days, standardized difference -0.74), and for active RSV (median 0 versus 14 days, standardized difference -0.97) than patients in Group 2. Co-infection with ≥2 conditions of interest (COVID-19, influenza, RSV) was found in 0.4% of Group 1 and 0.1% of Group 2 (standardized difference 0.07); the time from index date to diagnosis of co-infection among these patients was faster for Group 1 than Group 2 (median 0 versus 6 days, standardized difference -0.72).

### Use of treatments

Table 3 shows the use of treatments among all study patients and among the subset of patients with a diagnosis of active COVID-19, influenza, or RSV in the follow-up period. Compared with Group 2, Group 1 had higher use of a treatment for COVID-19, influenza, or RSV (7.4% versus 4.3%, standardized difference 0.14), and use of a treatment for influenza (1.6% versus 0.4%, standardized difference 0.12). Among patients with use of a treatment, patients in Group 1 had a faster time from index date to treatment of COVID-19, influenza, or RSV (median 1 versus 5 days, standardized difference -0.16) and to treatment of influenza (median 0 versus 3 days, standardized difference -0.63), but a longer time from index date to treatment for RSV (median 21 versus 16 days, standardized difference 0.25) than patients in Group 2.

Among patients with a diagnosis of active COVID-19, 18.2% of Group 1 and 15.9% of Group 2 used a treatment for COVID-19 (standardized difference 0.06) and the median time from index date to treatment was 1 versus 5 days (standardized difference -0.08). Among patients with a diagnosis of active influenza, Group 1 had numerically but not statistically higher use of a treatment for influenza than Group 2 (32.6% versus 30.9%, standardized difference 0.04) and a significantly faster time from index date to influenza treatment (median 0 versus 14 days, standardized difference -0.90). The number of patients with a diagnosis of active RSV and use of an RSV treatment was too small to evaluate.

## Discussion

We characterized the real-world use of tests and treatments for respiratory pathogens (SARS-CoV-2, influenza, and RSV) among US patients who presented with ILI in the outpatient setting and received SARS-CoV-2 testing with Xpert Xpress or a laboratory-based molecular test. Patients with Xpert Xpress testing (Group 1) had fewer claims for respiratory pathogen (SARS-CoV-2, influenza, and RSV) tests, a faster time to diagnosis of active respiratory infections (COVID-19, influenza, and RSV), and a faster time to treatment of COVID-19 and influenza than patients with laboratory-based molecular testing (Group 2).

With recent advancements in the availability of rapid, POC molecular multiplex testing for respiratory pathogens, this study provides context on the real-world utilization of respiratory virus tests for a large sample of patients across the US. Patients in Group 1 had different baseline characteristics than patients in Group 2, including more risk factors for ILI complications, a higher comorbidity burden, and higher distributions of patients <5 years or ≥65 years of age. Differences in patient characteristics may be due, in part, to the types of outpatient sites that have platforms available for POC testing as these platforms are more frequently found in EDs or large medical practices. Higher risk patients may seek care in the ED over primary care settings. As shown in this study, nearly one-third of patients with Xpert Xpress POC testing received their testing in the ED on the index date.

Although the objective of this analysis was to compare Xpert Xpress POC testing with laboratory-based molecular testing, we intentionally included all patients with outpatient Xpert Xpress testing (POC or laboratory-based) on the index date to be able to fully characterize outpatient Xpert Xpress use and avoid biasing the results to a subset of patients who only had POC testing. As expected, most patients (97%) with Xpert Xpress testing received POC only testing on the index date.

While the differences in patient characteristics between our study groups may be perceived as a limitation, we did not conduct multivariable analyses to control for these differences to preserve the aim of our study which was to assess real-world use patterns and outcomes with Xpert Xpress versus laboratory-based molecular testing. Statistical adjustment or propensity score matching to make the groups appear similar to each other would have negated the ability to characterize the actual utilization of tests and treatments across the entire study groups.

Despite having more risk factors, patients in Group 1 ended up having fewer claims for SARS-CoV-2, influenza, or RSV testing on the index date and through the end of the 90-day follow-up period than patients in Group 2. Among patients with an additional test for a respiratory pathogen after the index date, Group 1 had a longer time to additional test than Group 2. These findings are not surprising given that Xpert Xpress tests for multiple pathogens (SARS-CoV-2, influenza A, influenza B, with or without RSV) in one test which can reduce the need to administer multiple tests for single pathogens.

Notably, the higher frequency of claims for testing in Group 2 patients than Group 1 patients appeared to be driven by SARS-CoV-2 testing on the index date and not by additional testing for influenza or RSV. On the index date, 50% of patients in Group 2 had 2 or more claims for SARS-CoV-2 testing compared with 15% in Group 1. Possible explanations include patients with laboratory-based testing receiving SARS-CoV-2 molecular testing and diagnosis/treatment services at more than one site (e.g., physician office and then emergency department) on the index date, physicians ordering a laboratory-based reflex panel test after initial testing, or providers sending samples to different laboratories to increase chances of obtaining results as soon as possible. While these findings suggest that nearly one-half of patients who received laboratory-based molecular testing were re-tested for SARS-CoV-2 on the index date, further research is warranted to understand the clinical and administrative reasons behind these observations.

Consistent with previous studies [9, 10], the time from testing date to diagnosis of active COVID-19, active influenza, active RSV, and co-infection were faster for Group 1 than Group 2. A retrospective study of patients with ED visits at several US hospitals early in the COVID-19 pandemic found that use of Xpert Xpress SARS-CoV-2 tests instead of standard molecular tests resulted in faster time from test order to test result (median 1.9 versus 7.8 hours, p<0.001) and a higher frequency of availability of test results prior to ED departure (92% versus 51%, p<0.001) [9]. Another study showed a 70% reduction in time from sample collection to test result when Xpert Flu/RSV testing was conducted at POC sites instead of sending samples by courier to a central laboratory (median 1.3 versus 6.9 hours, p<0.001) [10].

Along with having a faster time to diagnosis, Group 1 had a faster time to treatment of any virus (COVID-19, influenza, or RSV) than Group 2. However, as roughly one-third of Group 1 testing occurred in the emergency setting, we cannot rule out the possibility that the faster time to diagnosis and faster time to treatment were related to more severely ill patients receiving the Xpert Xpress testing than laboratory-based molecular testing.

To further assess antiviral stewardship (optimize necessary use and reduce unnecessary use), the percentage of patients with treatment and time to treatment were evaluated among the subsets of patients with diagnosis of active COVID-19 or diagnosis of active influenza. Among patients with a diagnosis of active influenza, patients in Group 1 had numerically but not statistically higher use of an antiviral than patients in Group 2 and a significantly faster time from index date to treatment. These findings can be corroborated with previous studies that showed improved antiviral stewardship with use of rapid POC testing for influenza [10, 17]. In one study, use of Xpert Flu/RSV at POC instead of laboratory testing led to lower rates of under-treatment or over-treatment with antivirals than use of laboratory testing (10% versus 25%, p<0.001) [10]. In another study of patients with positive influenza tests in the ED, patients with rapid influenza PCR tests were more likely to be prescribed antivirals than patients with standard PCR testing (OR 4.92, 95% CI 2.13–11.34) [17]. A pre-post study after implementing rapid influenza PCR testing in an ED found lower rates of influenza empiric treatment among influenza-negative patients and higher rates of antiviral treatment and faster time to treatment among influenza-positive patients [18].

Among patients with a diagnosis of active COVID-19, the percentage of patients with a treatment for COVID-19 was numerically but not statistically higher and the time from index date to treatment was numerically, but not statistically, faster for Group 1 versus Group 2. The ability to interpret these COVID-19 treatment findings may be confounded by changes in availability of COVID-19 treatments and growth in scientific evidence about how to treat COVID-19 across the study follow-up timeframe (January 2021 through September 2022). Oral antiviral therapies (nirmatrelvir co-administered with ritonavir and molnupiravir) were not authorized by the FDA until December 2021 and thereafter required rationing for the most vulnerable patients due to short supply in periods of high demand [19].

### Limitations

Patients identified in Group 1 of this study were required to have Xpert Xpress testing, which may limit the ability to generalize the POC testing results to tests other than Xpert Xpress.

In addition, this study is subject to several limitations related to its retrospective design and data source type. Insurance claims were originally collected for billing and reimbursement and not for research. Procedures, services, or treatments can be missing from claims data if they were not billed to the insurance provider, or they were billed outside of the study timeframe. Although claims data capture treatments billed through prescription claims and tests and treatments administered in the outpatient setting, specific tests and treatments administered in the hospital setting may not be visible in claims data as they may be billed as a bundled payment under the overall hospital stay. Other data that may be missing from billing in claims data include SARS-CoV-2 or other respiratory pathogen tests self-administered at home or outside the physician office setting, SARS-CoV-2 or influenza vaccines administered at mass vaccination centers, and over-the-counter treatments.

Test result data were not available for this analysis. As a result, determination of diagnosis of COVID-19, influenza, and RSV relied on medical claims outside the laboratory setting with an active (non-historical) diagnosis code for the condition. Diagnosis was not able to be confirmed by the patient medical record. In some cases, claims may have been coded with a diagnosis code when the patient was being evaluated with the condition but not actually confirmed with the condition. While results from this study were based on relatively recent real-world data from 2021 and 2022, patterns of testing and treatment for influenza-like illnesses continue to evolve. With the emergence of new SARS-CoV-2 variants, physicians may prefer using specific molecular tests with newly emerged and currently circulating variant targets that may not be well-detected by rapid antigen tests [20]. Preferences for multiplex respiratory panel tests may increase, especially during times of year when multiple respiratory pathogens are circulating, as more POC and laboratory-based panel tests are developed and marketed to physicians.

Changes in health care policies after the declaration of the end of the federal COVID-19 PHE on May 11, 2023 [2, 21] may further impact the application of these results. Health insurance providers are no longer required to waive the costs of SARS-CoV-2 testing for patients [21], which may change patterns of frequency of testing, types of tests used, viruses tested, and the testing setting (home, POC, laboratory). While treatments for COVID-19 remain available, payment coverage of treatments may be dependent on the patient’s health plan [21]. With reductions in the frequency, metrics, and geographic areas of COVID-19 surveillance reporting [21], physicians may prefer panel tests with multiple pathogen targets and POC testing to facilitate a prompt and accurate diagnosis. As these and other factors may have impacted the testing and treatment landscape, further studies are warranted to assess changes in testing and treatment patterns of COVID-19 and other respiratory infections in the post-pandemic era.

## Conclusion

In this nationwide real-world study of molecular SARS-CoV-2 testing in the outpatient setting, patients with POC molecular multiplex testing had fewer claims for respiratory pathogen (SARS-CoV-2, influenza, and RSV) tests, a faster time to diagnosis of active respiratory infections (COVID-19, influenza, and RSV), and a faster time to treatment of COVID-19 and influenza than patients with laboratory-based molecular testing.

## Supporting information

S1 Table Procedure codes used to identify patients with a test for SARS-CoV-2 (± influenza or RSV) on the index date. (DOCX)

S2 Table Diagnosis codes for ILI symptoms and infections. (DOCX)

S3 Table Procedure codes used to identify tests for SARS-CoV-2, influenza, and/or RSV on the index date and in the follow-up period. (DOCX)

S4 Table Diagnosis codes for respiratory risk factors. (DOCX)

S5 Table Diagnosis and procedure codes for non-respiratory risk factors. (DOCX)

S6 Table Diagnosis codes for active COVID-19, influenza, and RSV. (DOCX)

S7 Table Treatments for COVID-19, influenza, and RSV. (DOCX)
