# Supplementary material for: Real-world use of multiplex point-of-care molecular testing or laboratory-based molecular testing for influenza-like illness in a 2021 to 2022 US outpatient sample
Source: PLoS One. 2024 Nov 11;19(11):e0313660. doi: 10.1371/journal.pone.0313660 (PMC11554232; doi:10.1371/journal.pone.0313660)
Supplement: S1 Table — (DOCX) [file pone.0313660.s001.docx]

# S1 Table. Procedure Codes Used to Identify Patients with a Test for SARS-CoV-2 (± Influenza or RSV) on the Index Date

| **Virus(es) Tested** | **Test Type** | **Procedure Codes** |
| --- | --- | --- |
| SARS-CoV-2 only | Antigen | CPT codes: 87426, 87811 |
|  | Molecular | CPT codes: 87635  HCPCS codes: U0001, U0003, U0005 |
|  | Unknown | HCPCS codes: U0002, U0004 |
| SARS-CoV-2 + influenza | Antigen | CPT codes: 87428 |
|  | Molecular | CPT codes: 87636, 0240U |
| SARS-CoV-2 + influenza + RSV | Molecular | CPT codes: 87637, 0241U |

CPT = Current Procedural Terminology, HCPCS = Healthcare Common Procedure Coding System, SARS-CoV-2 = severe acute respiratory syndrome coronavirus 2, RSV = respiratory syncytial virus
