# Supplementary material for: Real-world use of multiplex point-of-care molecular testing or laboratory-based molecular testing for influenza-like illness in a 2021 to 2022 US outpatient sample
Source: PLoS One. 2024 Nov 11;19(11):e0313660. doi: 10.1371/journal.pone.0313660 (PMC11554232; doi:10.1371/journal.pone.0313660)
Supplement: S2 Table — (DOCX) [file pone.0313660.s002.docx]

# S2 Table. Diagnosis Codes for ILI Symptoms and Infections

| **ICD-10 Diagnosis Code** | **Description** |
| --- | --- |
| Z8616 | Personal history of COVID-19 |
| J1282 | Pneumonia due to coronavirus disease 2019 |
| U071 | COVID-19 |
| U099 | Post COVID-19 condition, unspecified |
| B974 | Respiratory syncytial virus as the cause of diseases classified elsewhere |
| J121 | Respiratory syncytial virus pneumonia |
| J205 | Acute bronchitis due to respiratory syncytial virus |
| J210 | Acute bronchiolitis due to respiratory syncytial virus |
| J09X1 | Influenza due to identified novel influenza A virus with pneumonia |
| J09X2 | Influenza due to identified novel influenza A virus with other respiratory manifestations |
| J09X3 | Influenza due to identified novel influenza A virus with gastrointestinal manifestations |
| J09X9 | Influenza due to identified novel influenza A virus with other manifestations |
| J1000 | Influenza due to other identified influenza virus with unspecified type of pneumonia |
| J1001 | Influenza due to other identified influenza virus with the same other identified influenza virus pneumonia |
| J1008 | Influenza due to other identified influenza virus with other specified pneumonia |
| J101 | Influenza due to other identified influenza virus with other respiratory manifestations |
| J102 | Influenza due to other identified influenza virus with gastrointestinal manifestations |
| J1081 | Influenza due to other identified influenza virus with encephalopathy |
| J1082 | Influenza due to other identified influenza virus with myocarditis |
| J1083 | Influenza due to other identified influenza virus with otitis media |
| J1089 | Influenza due to other identified influenza virus with other manifestations |
| J1100 | Influenza due to unidentified influenza virus with unspecified type of pneumonia |
| J1108 | Influenza due to unidentified influenza virus with specified pneumonia |
| J111 | Influenza due to unidentified influenza virus with other respiratory manifestations |
| J112 | Influenza due to unidentified influenza virus with gastrointestinal manifestations |
| J1181 | Influenza due to unidentified influenza virus with encephalopathy |
| J1182 | Influenza due to unidentified influenza virus with myocarditis |
| J1183 | Influenza due to unidentified influenza virus with otitis media |
| J1189 | Influenza due to unidentified influenza virus with other manifestations |
| R05 | Cough |
| R051 | Acute cough |
| R052 | Subacute cough |
| R053 | Chronic cough |
| R058 | Other specified cough |
| R059 | Cough, unspecified |
| R0602 | Shortness of breath |
| R0600 | Dyspnea, unspecified |
| R509 | Fever, unspecified |
| J1289 | Other viral pneumonia |
| J129 | Viral pneumonia, unspecified |
| J208 | Acute bronchitis due to other specified organisms |
| J209 | Acute bronchitis, unspecified |
| J22 | Unspecified acute lower respiratory infection |
| J40 | Bronchitis, not specified as acute or chronic |
| J80 | Acute respiratory distress syndrome |
| J9600 | Acute respiratory failure, unspecified whether with hypoxia or hypercapnia |
| J9601 | Acute respiratory failure with hypoxia |
| J9602 | Acute respiratory failure with hypercapnia |
| J988 | Other specified respiratory disorders |
| J989 | Respiratory disorder, unspecified |
| J069 | Acute upper respiratory infection, unspecified |
| J218 | Acute bronchiolitis due to other specified organisms |
| J219 | Acute bronchiolitis, unspecified |
| B9721 | SARS-associated coronavirus as the cause of diseases classified elsewhere |
| B9729 | Other coronavirus as the cause of diseases classified elsewhere |
| J00 | Acute nasopharyngitis [common cold] |
| J1281 | Pneumonia due to SARS-associated coronavirus |
| J168 | Pneumonia due to other specified infectious organisms |
| J17 | Pneumonia in diseases classified elsewhere |
| J180 | Bronchopneumonia, unspecified organism |
| J181 | Lobar pneumonia, unspecified organism |
| J182 | Hypostatic pneumonia, unspecified organism |
| J188 | Other pneumonia, unspecified organism |
| J189 | Pneumonia, unspecified organism |
| R430 | Anosmia |
| R438 | Other disturbances of smell and taste |
| R439 | Unspecified disturbances of smell and taste |
| J029 | Acute pharyngitis, unspecified |
| R519 | Headache, unspecified |

ICD-10 = International Classification of Diseases 10th Revision, ILI = influenza-like illness
