# Supplementary material for: Real-world use of multiplex point-of-care molecular testing or laboratory-based molecular testing for influenza-like illness in a 2021 to 2022 US outpatient sample
Source: PLoS One. 2024 Nov 11;19(11):e0313660. doi: 10.1371/journal.pone.0313660 (PMC11554232; doi:10.1371/journal.pone.0313660)
Supplement: S4 Table — (DOCX) [file pone.0313660.s004.docx]

# S4 Table. Diagnosis Codes for Respiratory Risk Factors

| **Risk Factor** | **Code Type** | **Codes** |
| --- | --- | --- |
| Asthma | ICD-10 diagnosis | J4520, J4521, J4522, J4530, J4531, J4532, J4540, J4541, J4542, J4550, J4551, J4552, J45901, J45902, J45909, J45990, J45991, J45998, J8283 |
| Chronic lung disease | ICD-10 diagnosis | B250, B4481, B583, D860, D862, E840, J17, J410, J411, J418, J42, J430, J431, J432, J438, J439, J440, J441, J449, J60, J61, J620, J628, J630, J631, J632, J633, J634, J635, J636, J64, J65, J660, J661, J662, J668, J670, J671, J672, J673, J674, J675, J676, J677, J678, J679, J680, J681, J682, J684, J688, J689, J690, ,J691, J698, J701, J703, J704, J705, J708, J709, J811, J82, J8281, J8289, J8401, J8402, J8403, J8409, J8410, J84111, J84112, J84113, J84115, J84116, J84117, J8417, J84170, J84178, J842, J8481, J8482, J8483, J84841, J84842, J84843, J84848, J8489, J849, J850, J851, J852, J860, J869, J90, J910, J918, J920, J929, J930, J9311, J9312, J9381, J9383, J939, J940, J941, J942, J948, J949, J953, J954, J95822, J9589, J9610, J9611, J9612, J9620, J9621, J9622, J9690, J9691, J9692, J9819, J983, J984, M0510 M05111, M05112, M05119, M05121, M05122, M05129, M05131, M05132, M05139, M05141, M05142, M05149, M05151, M05152, M05159, M05161, M05162, M05169, M05171, M05172, M05179, M0519, M301, M3213, M3481, M3502, J40 |

ICD-10 = International Classification of Diseases 10th Revision
