# Supplementary material for: Real-world use of multiplex point-of-care molecular testing or laboratory-based molecular testing for influenza-like illness in a 2021 to 2022 US outpatient sample
Source: PLoS One. 2024 Nov 11;19(11):e0313660. doi: 10.1371/journal.pone.0313660 (PMC11554232; doi:10.1371/journal.pone.0313660)
Supplement: S5 Table — (DOCX) [file pone.0313660.s005.docx]

# S5 Table. Diagnosis and Procedure Codes for Non-Respiratory Risk Factors

| **Risk Factor** | **Code Type** | **Codes** |
| --- | --- | --- |
| Diabetes | ICD-10 diagnosis | E0800, E0801, E0810, E0811, E0821, E0822, E0829, E08311, E08319, E083211, E083212, E083213, E083219, E083291, E083292, E083293, E083299, E083311, E083312, E083313, E083319, E083391, E083392, E083393, E083399, E083411, E083412, E083413, E083419, E083491, E083492, E083493, E083499, E083511, E083512, E083513, E083519, E083521, E083522, E083523, E083529, E083531, E083532, E083533, E083539, E083541, E083542, E083543, E083549, E083551, E083552, E083553, E083559, E083591, E083592, E083593, E083599, E0836, E0837X1, E0837X2, E0837X3, E0837X9, E0839, E0840, E0841, E0842, E0843, E0844, E0849, E0851, E0852, E0859, E08610, E08618, E08620, E08621, E08622, E08628, E08630, E08638, E08641, E08649, E0865, E0869, E088, E089, E0900, E0901, E0910, E0911, E0921, E0922, E0929, E09311, E09319, E093211, E093212, E093213, E093219, E093291, E093292, E093293, E093299, E093311, E093312, E093313, E093319, E093391, E093392, E093393, E093399, E093411, E093412, E093413, E093419, E093491, E093492, E093493, E093499, E093511, E093512, E093513, E093519, E093521, E093522, E093523, E093529, E093531, E093532, E093533, E093539, E093541, E093542, E093543, E093549, E093551, E093552, E093553, E093559, E093591, E093592, E093593, E093599, E0936, E0937X1, E0937X2, E0937X3, E0937X9, E0939, E0940, E0941, E0942, E0943, E0944, E0949, E0951, E0952, E0959, E09610, E09618, E09620, E09621, E09622, E09628, E09630, E09638, E09641, E09649, E0965, E0969, E098, E099, E1010, E1011, E1021, E1022, E1029, E10311, E10319, E103211, E103212, E103213, E103219, E103291, E103292, E103293, E103299, E103311, E103312, E103313, E103319, E103391, E103392, E103393, E103399, E103411, E103412, E103413, E103419, E103491, E103492, E103493, E103499, E103511, E103512, E103513, E103519, E103521, E103522, E103523, E103529, E103531, E103532, E103533, E103539, E103541, E103542, E103543, E103549, E103551, E103552, E103553, E103559, E103591, E103592, E103593, E103599, E1036, E1037X1, E1037X2, E1037X3, E1037X9, E1039, E1040, E1041, E1042, E1043, E1044, E1049, E1051, E1052, E1059, E10610, E10618, E10620, E10621, E10622, E10628, E10630, E10638, E10641, E10649, E1065, E1069, E108, E109, E1100, E1101, E1110, E1111, E1121, E1122, E1129, E11311, E11319, E113211, E113212, E113213, E113219, E113291, E113292, E113293, E113299, E113311, E113312, E113313, E113319, E113391, E113392, E113393, E113399, E113411, E113412, E113413, E113419, E113491, E113492, E113493, E113499, E113511, E113512, E113513, E113519, E113521, E113522, E113523, E113529, E113531, E113532, E113533, E113539, E113541, E113542, E113543, E113549, E113551, E113552, E113553, E113559, E113591, E113592, E113593, E113599, E1136, E1137X1, E1137X2, E1137X3, E1137X9, E1139, E1140, E1141, E1142, E1143, E1144, E1149, E1151, E1152, E1159, E11610, E11618, E11620, E11621, E11622, E11628, E11630, E11638, E11641, E11649, E1165, E1169, E118, E119, E1300, E1301, E1310, E1311, E1321, E1322, E1329, E13311, E13319, E133211, E133212, E133213, E133219, E133291, E133292, E133293, E133299, E133311, E133312, E133313, E133319, E133391, E133392, E133393, E133399, E133411, E133412, E133413, E133419, E133491, E133492, E133493, E133499, E133511, E133512, E133513, E133519, E133521, E133522, E133523, E133529, E133531, E133532, E133533, E133539, E133541, E133542, E133543, E133549, E133551, E133552, E133553, E133559, E133591, E133592, E133593, E133599, E1336, E1337X1, E1337X2, E1337X3, E1337X9, E1339, E1340, E1341, E1342, E1343, E1344, E1349, E1351, E1352, E1359, E13610, E13618, E13620, E13621, E13622, E13628, E13630, E13638, E13641, E13649, E1365, E1369, E138, E139, O24011, O24012, O24013, O24019, O2402, O2403, O24111, O24112, O24113, O24119, O2412, O2413, O24311, O24312, O24313, O24319, O2432, O2433, O24811, O24812, O24813, O24819, O2482, O2483, O24911, O24912, O24913, O24919, O2492, O2493, O99810, O99814, O99815, T383X6A, T383X6D, T383X6S, T85614A, T85614D, T85614S, T85624A, T85624D, T85624S, T85633A, T85633D, T85633S, T85694A, T85694D, T85694S, T8572XA, T8572XD, T8572XS, Z4681, Z794, Z8631, Z9641 |
|  | CPT | 95250, 95251 |
|  | HCPCS | G9147, S3000, S9455, S9460, S9465 |
| Neurologic conditions | ICD-10 diagnosis | F70, F71, F72, F73, F78, F78A1, F78A9, F79, F800, F801, F802, F804, F8081, F8082, F8089, F809, F810, F812, F8181, F8189, F819, F82, F840, F842, F843, F845, F848, F849, F88, F89, G000, G001, G002, G003, G008, G009, G01, G02, G030, G031, G032, G038, G039, G0400, G0401, G0402, G041, G042, G0430, G0431, G0432, G0439, G0481, G0482, G0489, G0490, G0491, G053, G054, G060, G061, G062, G07, G08, G09, G10, G110, G111, G1110, G1111, G1119, G112, G113, G114, G118, G119, G120, G121, G1220, G1221, G1222, G1223, G1224, G1225, G1229, G128, G129, G130, G131, G132, G138, G14, G20, G210, G2111, G2119, G212, G213, G214, G218, G219, G230, G231, G232, G238, G239, G2401, G2402, G2409, G241, G242, G243, G244, G245, G248, G249, G250, G251, G252, G253, G254, G255, G2561, G2569, G2570, G2571, G2579, G2581, G2582, G2583, G2589, G259, G26, G300, G301, G308, G309, G3101, G3109, G311, G312, G3181, G3182, G3183, G3184, G3185, G3189, G319, G320, G3281, G3289, G35, G360, G361, G368, G369, G370, G371, G372, G373, G374, G375, G378, G379, G40001, G40009, G40011, G40019, G40101, G40109, G40111, G40119, G40201, G40209, G40211, G40219, G40301, G40309, G40311, G40319, G40401, G40409, G40411, G40419, G4042, G40501, G40509, G40801, G40802, G40803, G40804, G40811, G40812, G40813, G40814, G40821, G40822, G40823, G40824, G40833, G40834, G4089, G40901, G40909, G40911, G40919, G40A01, G40A09, G40A11, G40A19, G40B01, G40B09, G40B11, G40B19, G43001, G43009, G43011, G43019, G43101, G43109, G43111, G43119, G43401, G43409, G43411, G43419, G43501, G43509, G43511, G43519, G43601, G43609, G43611, G43619, G43701, G43709, G43711, G43719, G43801, G43809, G43811, G43819, G43821, G43829, G43831, G43839, G43901, G43909, G43911, G43919, G43A0, G43A1, G43B0, G43B1, G43C0, G43C1, G43D0, G43D1, G44001, G44009, G44011, G44019, G44021, G44029, G44031, G44039, G44041, G44049, G44051, G44059, G44091, G44099, G441, G44201, G44209, G44211, G44219, G44221, G44229, G44301, G44309, G44311, G44319, G44321, G44329, G4440, G4441, G4451, G4452, G4453, G4459, G4481, G4482, G4483, G4484, G4485, G4486, G4489, G450, G451, G452, G453, G454, G458, G459, G460, G461, G462, G463, G464, G465, G466, G467, G468, G4700, G4701, G4709, G4710, G4711, G4712, G4713, G4714, G4719, G4720, G4721, G4722, G4723, G4724, G4725, G4726, G4727, G4729, G4730, G4731, G4732, G4733, G4734, G4735, G4736, G4737, G4739, G47411, G47419, G47421, G47429, G4750, G4751, G4752, G4753, G4754, G4759, G4761, G4762, G4763, G4769, G478, G479, G500, G501, G508, G509, G510, G511, G512, G513, G5131, G5132, G5133, G5139, G514, G518, G519, G520, G521, G522, G523, G527, G528, G529, G53, G540, G541, G542, G543, G544, G545, G546, G547, G548, G549, G55, G5600, G5601, G5602, G5603, G5610, G5611, G5612, G5613, G5620, G5621, G5622, G5623, G5630, G5631, G5632, G5633, G5640, G5641, G5642, G5643, G5680, G5681, G5682, G5683, G5690, G5691, G5692, G5693, G5700, G5701, G5702, G5703, G5710, G5711, G5712, G5713, G5720, G5721, G5722, G5723, G5730, G5731, G5732, G5733, G5740, G5741, G5742, G5743, G5750, G5751, G5752, G5753, G5760, G5761, G5762, G5763, G5770, G5771, G5772, G5773, G5780, G5781, G5782, G5783, G5790, G5791, G5792, G5793, G580, G587, G588, G589, G59, G600, G601, G602, G603, G608, G609, G610, G611, G6181, G6182, G6189, G619, G620, G621, G622, G6281, G6282, G6289, G629, G63, G64, G650, G651, G652, G7000, G7001, G701, G702, G7080, G7081, G7089, G709, G710, G7100, G7101, G7102, G7103, G71031, G71032, G71033, G71034, G710340, G710341, G710342, G710349, G71035, G71038, G71039, G7109, G7111, G7112, G7113, G7114, G7119, G712, G7120, G7121, G71220, G71228, G7129, G713, G718, G719, G720, G721, G722, G723, G7241, G7249, G7281, G7289, G729, G731, G733, G737, G800, G801, G802, G803, G804, G808, G809, G8100, G8101, G8102, G8103, G8104, G8110, G8111, G8112, G8113, G8114, G8190, G8191, G8192, G8193, G8194, G8220, G8221, G8222, G8250, G8251, G8252, G8253, G8254, G830, G8310, G8311, G8312, G8313, G8314, G8320, G8321, G8322, G8323, G8324, G8330, G8331, G8332, G8333, G8334, G834, G835, G8381, G8382, G8383, G8384, G8389, G839, G890, G8911, G8912, G8918, G8921, G8922, G8928, G8929, G893, G894, G9001, G9009, G901, G902, G903, G904, G9050, G90511, G90512, G90513, G90519, G90521, G90522, G90523, G90529, G9059, G908, G909, G90A, G910, G911, G912, G913, G914, G918, G919, G9200, G9201, G9202, G9203, G9204, G9205, G928, G929, G930, G931, G932, G933, G9331, G9332, G9339, G9340, G9341, G9349, G935, G936, G937, G9381, G9382, G9389, G939, G94, G950, G9511, G9519, G9520, G9529, G9581, G9589, G959, G960, G9600, G9601, G9602, G9608, G9609, G9611, G9612, G9619, G96191, G96198, G968, G96810, G96811, G96819, G9689, G969, G970, G971, G972, G9731, G9732, G9741, G9748, G9749, G9751, G9752, G9761, G9762, G9763, G9764, G9781, G9782, G9783, G9784, G980, G988, G990, G992, G998 |
| Immunocompromised status | ICD-10 diagnosis | B20, B9735, D469, D46Z, D693, D700, D701, D704, D71, D720, D730, D735, D800, D801, D802, D803, D804, D805, D806, D807, D808, D809, D810, D811, D812, D813, D8131, D814, D815, D816, D817, D81810, D81818, D81819, D8189, D819, D820, D821, D822, D823, D824, D828, D829, D830, D831, D832, D838, D839, D840, D841, D848, D8481, D84821, D84822, D8489, D849, D8989, D899, G379, O98711, O98712, O98713, O98719, O9872, O9873, Q8901, Z21, Z717, Z9081 |
| Transplant | ICD-10 diagnosis | Z4821, Z4822, Z4823, Z4824, Z48280, Z48288, Z48290, Z48298, C802, D47Z1, I25750, I25751, I25758, I25759, I25760, I25761, I25768, I25769, I25811, I25812, T8600, T8601, T8602, T8603, T8609, T8610, T8611, T8612, T8613, T8619, T8620, T8621, T8622, T8623, T86290, T86298, T8630, T8631, T8632, T8633, T8639, T8640, T8641, T8642, T8643, T8649, T865, T86810, T86811, T86812, T86818, T86819, T86850, T86851, T86852, T86858, T86859, T86890, T86891, T86892, T86898, T86899, T8690, T8691, T8692, T8693, T8699, Y830, Z9885, Z940, Z941, Z942, Z943, Z944, Z946, Z9481, Z9482, Z9483, Z9484, Z9489, Z949 |
| Cancer | ICD-10 diagnosis | C494, C762, C310, C311, C312, C313, C318, C319, C7400, C7401, C7402, C7410, C7411, C7412, C7490, C7491, C7492, C7970, C7971, C7972, C210, C211, C212, C218, C221, C240, C241, C248, C249, C4000, C4001, C4002, C4010, C4011, C4012, C4020, C4021, C4022, C4030, C4031, C4032, C410, C411, C412, C413, C414, C7951, C7B03, C7952, D474, D7581, C4080, C4081, C4082, C4090, C4091, C4092, C419, C710, C711, C712, C713, C714, C715, C716, C717, C718, C719, C7931, C50011, C50012, C50019, C50021, C50022, C50029, C50111, C50112, C50119, C50121, C50122, C50129, C50211, C50212, C50219, C50221, C50222, C50229, C50311, C50312, C50319, C50321, C50322, C50329, C50411, C50412, C50419, C50421, C50422, C50429, C50511, C50512, C50519, C50521, C50522, C50529, C50611, C50612, C50619, C50621, C50622, C50629, C50811, C50812, C50819, C50821, C50822, C50829, C50911, C50912, C50919, C50921, C50922, C50929, C7981, C847A, C512, C180, C181, C182, C183, C184, C185, C186, C187, C188, C189, C49A4, C7A020, C7A021, C7A022, C7A023, C7A024, C7A025, C7A029, C7A096, C785, C19, C7220, C7221, C7222, C7230, C7231, C7232, C7240, C7241, C7242, C7250, C7259, C729, C800, C755, C758, C759, C541, C6300, C6301, C6302, C153, C154, C155, C158, C159, C49A1, C8109, C8119, C8129, C8139, C8149, C8179, C8199, C8209, C8219, C8229, C8239, C8249, C8259, C8269, C8289, C8299, C8309, C8319, C8339, C8359, C8379, C8389, C8399, C8409, C8419, C8449, C8469, C8479, C8499, C84A9, C84Z9, C8519, C8529, C8589, C8599, C860, C884, C6900, C6901, C6902, C6910, C6911, C6912, C6920, C6921, C6922, C6930, C6931, C6932, C6940, C6941, C6942, C6950, C6951, C6952, C6960, C6961, C6962, C6980, C6981, C6982, C6990, C6991, C6992, C5700, C5701, C5702, C23, C260, C269, C464, C49A0, C49A9, C7880, C7889, C577, C578, C579, C609, C637, C638, C639, C7982, C490, C760, C380, C388, C12, C130, C131, C132, C138, C139, C7A095, C460, C641, C642, C649, C7900, C7901, C7902, C510, C511, C322, C323, C328, C329, C220, C222, C223, C224, C227, C228, C229, C787, C7B02, C4920, C4921, C4922, C3400, C3401, C3402, C3410, C3411, C3412, C342, C3430, C3431, C3432, C3480, C3481, C3482, C3490, C3491, C3492, C4650, C4651, C4652, C7800, C7801, C7802, C7A090, C463, C770, C771, C772, C773, C774, C775, C778, C779, C7B01, C8101, C8102, C8103, C8104, C8105, C8106, C8108, C8111, C8112, C8113, C8114, C8115, C8116, C8118, C8121, C8122, C8123, C8124, C8125, C8126, C8128, C8131, C8132, C8133, C8134, C8135, C8136, C8138, C8141, C8142, C8143, C8144, C8145, C8146, C8148, C8171, C8172, C8173, C8174, C8175, C8176, C8178, C8191, C8192, C8193, C8194, C8195, C8196, C8198, C8201, C8202, C8203, C8204, C8205, C8206, C8208, C8211, C8212, C8213, C8214, C8215, C8216, C8218, C8221, C8222, C8223, C8224, C8225, C8226, C8228, C8231, C8232, C8233, C8234, C8235, C8236, C8238, C8241, C8242, C8243, C8244, C8245, C8246, C8248, C8251, C8252, C8253, C8254, C8255, C8256, C8258, C8261, C8262, C8263, C8264, C8265, C8266, C8268, C8281, C8282, C8283, C8284, C8285, C8286, C8288, C8291, C8292, C8293, C8294, C8295, C8296, C8298, C8301, C8302, C8303, C8304, C8305, C8306, C8308, C8311, C8312, C8313, C8314, C8315, C8316, C8318, C8331, C8332, C8333, C8334, C8335, C8336, C8338, C8351, C8352, C8353, C8354, C8355, C8356, C8358, C8371, C8372, C8373, C8374, C8375, C8376, C8378, C8381, C8382, C8383, C8384, C8385, C8386, C8388, C8391, C8392, C8393, C8394, C8395, C8396, C8398, C8401, C8402, C8403, C8404, C8405, C8406, C8408, C8411, C8412, C8413, C8414, C8415, C8416, C8418, C8441, C8442, C8443, C8444, C8445, C8446, C8448, C8461, C8462, C8463, C8464, C8465, C8466, C8468, C8471, C8472, C8473, C8474, C8475, C8476, C8478, C8491, C8492, C8493, C8494, C8495, C8496, C8498, C84A1, C84A2, C84A3, C84A4, C84A5, C84A6, C84A8, C84Z1, C84Z2, C84Z3, C84Z4, C84Z5, C84Z6, C84Z8, C8511, C8512, C8513, C8514, C8515, C8516, C8518, C8521, C8522, C8523, C8524, C8525, C8526, C8528, C8581, C8582, C8583, C8584, C8585, C8586, C8588, C8591, C8592, C8593, C8594, C8595, C8596, C8598, C381, C382, C383, C781, C700, C701, C709, C7932, C4A0, C4A10, C4A111, C4A112, C4A121, C4A122, C4A20, C4A21, C4A22, C4A30, C4A31, C4A39, C4A4, C4A51, C4A52, C4A59, C4A60, C4A61, C4A62, C4A70, C4A71, C4A72, C4A8, C4A9, C7B1, C301, C300, C109, C110, C111, C112, C113, C118, C119, C7940, C7949, C754, C024, C030, C031, C039, C040, C041, C048, C049, C050, C051, C052, C058, C059, C060, C061, C062, C0680, C0689, C069, C462, C090, C091, C100, C101, C102, C103, C104, C108, C142, C148, C498, C499, C561, C562, C563, C569, C7960, C7961, C7962, C7963, C250, C251, C252, C253, C254, C257, C258, C259, C750, C495, C763, C601, C602, C608, C452, C470, C4710, C4711, C4712, C4720, C4721, C4722, C473, C474, C475, C476, C478, C479, C451, C480, C481, C482, C488, C786, C7B04, C140, C320, C321, C753, C751, C752, C58, C384, C450, C457, C459, C782, C600, C61, C20, C49A5, C7A026, C651, C652, C659, C7A093, C390, C399, C7830, C7839, C07, C080, C081, C089, C098, C430, C4310, C43111, C43112, C43121, C43122, C4320, C4321, C4322, C4330, C4331, C4339, C434, C4351, C4352, C4359, C4360, C4361, C4362, C4370, C4371, C4372, C438, C439, C792, C866, C170, C171, C172, C173, C178, C179, C49A3, C784, C7A010, C7A011, C7A012, C7A019, C7A094, C883, C6310, C6311, C6312, C720, C721, C261, C8107, C8117, C8127, C8137, C8147, C8177, C8197, C8207, C8217, C8227, C8237, C8247, C8257, C8267, C8287, C8297, C8307, C8317, C8337, C8357, C8377, C8387, C8397, C8407, C8417, C8447, C8467, C8477, C8497, C84A7, C84Z7, C8517, C8527, C8587, C8597, C160, C161, C162, C163, C164, C165, C166, C168, C169, C49A2, C7A092, C8100, C8110, C8120, C8130, C8140, C8170, C8190, C8200, C8210, C8220, C8230, C8240, C8250, C8260, C8280, C8290, C8300, C8310, C8330, C8350, C8370, C8380, C8390, C8410, C8440, C8460, C8470, C8490, C84A0, C84Z0, C8510, C8520, C8580, C8590, C861, C862, C863, C864, C865, C880, C882, C888, C889, C9000, C9001, C9002, C9010, C9011, C9012, C9100, C9101, C9102, C9110, C9111, C9112, C9130, C9131, C9132, C9140, C9141, C9142, C9150, C9151, C9152, C9160, C9161, C9162, C9190, C9191, C9192, C91A0, C91A1, C91A2, C91Z0, C91Z1, C91Z2, C9200, C9201, C9202, C9210, C9211, C9212, C9220, C9221, C9222, C9230, C9231, C9232, C9240, C9241, C9242, C9250, C9251, C9252, C9260, C9261, C9262, C9290, C9291, C9292, C92A0, C92A1, C92A2, C92Z0, C92Z1, C92Z2, C9300, C9301, C9302, C9310, C9311, C9312, C9330, C9331, C9332, C9390, C9391, C9392, C93Z0, C93Z1, C93Z2, C9400, C9401, C9402, C9420, C9421, C9422, C9430, C9431, C9432, C9440, C9441, C9442, C946, C9480, C9481, C9482, C9500, C9501, C9502, C9510, C9511, C9512, C9590, C9591, C9592, C960, C9621, C964, C965, C966, C969, C96A, C96Z, D45, D460, D461, D4620, D4621, D4622, D464, D469, D46A, D46B, D46C, D46Z, D4702, D471, D472, D473, D47Z1, C6200, C6201, C6202, C6210, C6211, C6212, C6290, C6291, C6292, C493, C761, C37, C7A091, C73, C01, C020, C021, C022, C023, C028, C029, C099, C33, C802, C496, C461, C467, C469, C768, C7989, C799, C7A00, C7A098, C7A1, C7A8, C7B00, C7B09, C7B8, C801, C8400, C9020, C9021, C9022, C9030, C9031, C9032, C9620, C9622, C9629, M360, C4910, C4911, C4912, C7640, C7641, C7642, C7650, C7651, C7652, C661, C662, C669, C676, C680, C681, C688, C689, C670, C671, C672, C673, C674, C675, C677, C678, C679, C7911, C7910, C7919, C5710, C5711, C5712, C5720, C5721, C5722, C573, C574, C530, C531, C538, C539, C540, C542, C543, C548, C549, C55, C52, C518, C519 |
| Tuberculosis | ICD-10 diagnosis | A150, A154, A155, A156, A157, A158, A159, A170, A171, A1781, A1782, A1783, A1789, A179, A1801, A1802, A1803, A1809, A1810, A1811, A1812, A1813, A1814, A1815, A1816, A1817, A1818, A182, A1831, A1832, A1839, A184, A1850, A1851, A1852, A1853, A1854, A1859, A186, A187, A1881, A1882, A1883, A1884, A1885, A1889, A190, A191, A192, A198, A199, B900, B901, B902, B908, B909, J65, O98011, O98012, O98013, O98019, O9802, O9803, Z227 |
| Kidney disease | ICD-10 diagnosis | A1811, A5275, A5421, D631, E0821, E0822, E0829, E0921, E0922, E0929, E1021, E1022, E1029, E1121, E1122, E1129, E1321, E1322, E1329, I120, I129, I130, I1310, I1311, I132, I7581, I953, M1030, M10311, M10312, M10319, M10321, M10322, M10329, M10331, M10332, M10339, M10341, M10342, M10349, M10351, M10352, M10359, M10361, M10362, M10369, M10371, M10372, M10379, M1038, M1039, M1A30X0, M1A30X1, M1A3110, M1A3111, M1A3120, M1A3121, M1A3190, M1A3191, M1A3210, M1A3211, M1A3220, M1A3221, M1A3290, M1A3291, M1A3310, M1A3311, M1A3320, M1A3321, M1A3390, M1A3391, M1A3410, M1A3411, M1A3420, M1A3421, M1A3490, M1A3491, M1A3510, M1A3511, M1A3520, M1A3521, M1A3590, M1A3591, M1A3610, M1A3611, M1A3620, M1A3621, M1A3690, M1A3691, M1A3710, M1A3711, M1A3720, M1A3721, M1A3790, M1A3791, M1A38X0, M1A38X1, M1A39X0, M1A39X1, N000, N001, N002, N003, N004, N005, N006, N007, N008, N009, N010, N011, N012, N013, N014, N015, N016, N017, N018, N019, N020, N021, N022, N023, N024, N025, N026, N027, N028, N029, N030, N031, N032, N033, N034, N035, N036, N037, N038, N039, N040, N041, N042, N043, N044, N045, N046, N047, N048, N049, N050, N051, N052, N053, N054, N055, N056, N057, N058, N059, N060, N061, N062, N063, N064, N065, N066, N067, N068, N069, N070, N071, N072, N073, N074, N075, N076, N077, N078, N079, N08, N10, N110, N111, N118, N119, N12, N131, N132, N1330, N1339, N136, N1370, N1371, N13721, N13722, N13729, N13731, N13732, N13739, N138, N139, N140, N141, N1411, N1419, N142, N143, N144, N150, N151, N158, N159, N16, N170, N171, N172, N178, N179, N181, N182, N183, N1830, N1831, N1832, N184, N185, N186, N189, N19, N200, N202, N250, N251, N2581, N2589, N259, N261, N262, N269, N270, N271, N279, N280, N281, N2881, N2889, N289, N29, N990, O10211, O10212, O10213, O10219, O1022, O1023, O10311, O10312, O10313, O10319, O1032, O1033, O2300, O2301, O2302, O2303, O26831, O26832, O26833, O26839, O8621, O904, Q600, Q601, Q602, Q603, Q604, Q605, Q606, Q6100, Q6101, Q6102, Q6111, Q6119, Q612, Q613, Q614, Q615, Q618, Q619, Q620, Q630, Q631, Q632, Q633, Q638, Q639, R8279, R8281, R8289, R82991, R82992, R82993, R82994, R880, R93421, R93422, R93429, R944, S37001A, S37001D, S37001S, S37002A, S37002D, S37002S, S37009A, S37009D, S37009S, S37011A, S37011D, S37011S, S37012A, S37012D, S37012S, S37019A, S37019D, S37019S, S37021A, S37021D, S37021S, S37022A, S37022D, S37022S, S37029A, S37029D, S37029S, S37031A, S37031D, S37031S, S37032A, S37032D, S37032S, S37039A, S37039D, S37039S, S37041A, S37041D, S37041S, S37042A, S37042D, S37042S, S37049A, S37049D, S37049S, S37051A, S37051D, S37051S, S37052A, S37052D, S37052S, S37059A, S37059D, S37059S, S37061A, S37061D, S37061S, S37062A, S37062D, S37062S, S37069A, S37069D, S37069S, S37091A, S37091D, S37091S, S37092A, S37092D, S37092S, S37099A, S37099D, S37099S, T81502A, T81502A, T81502D, T81502D, T81502S, T81502S, T81512A, T81512A, T81512D, T81512D, T81512S, T81512S, T81522A, T81522A, T81522D, T81522D, T81522S, T81522S, T81532A, T81532A, T81532D, T81532D, T81532S, T81532S, T81592A, T81592A, T81592D, T81592D, T81592S, T81592S, T8241XA, T8241XD, T8241XS, T8242XA, T8242XD, T8242XS, T8243XA, T8243XD, T8243XS, T8249XA, T8249XD, T8249XS, T85611A, T85611D, T85611S, T85621A, T85621D, T85621S, T85631A, T85631D, T85631S, T85691A, T85691D, T85691S, T8571XA, T8571XD, T8571XS, T8610, T8611, T8612, T8613, T8619, Y622, Y622, Y841, Y841, Z4822, Z4901, Z4902, Z4931, Z4932, Z905, Z9115, Z91A5, Z940, Z992 |
| Liver disease | ICD-10 diagnosis | A064, A5145, A5274, B0081, B150, B159, B160, B161, B162, B169, B1710, B1711, B172, B178, B179, B180, B181, B182, B188, B189, B190, B1910, B1911, B1920, B1921, B199, B251, B2681, B581, B670, B675, B678, B942, E8021, I81, I820, I8500, I8501, I8510, I8511, I864, K652, K700, K7010, K7011, K702, K7030, K7031, K7040, K7041, K709, K710, K7110, K7111, K712, K713, K714, K7150, K7151, K716, K717, K718, K719, K7200, K7201, K7210, K7211, K7290, K7291, K730, K731, K732, K738, K739, K740, K7400, K7401, K7402, K741, K742, K743, K744, K745, K7460, K7469, K750, K751, K752, K753, K754, K7581, K7589, K759, K760, K761, K762, K763, K765, K766, K767, K7681, K7682, K7689, K769, K77, K835, K838, K839, K87, K9182, K9183, O26611, O26612, O26613, O26619, O2662, O2663, O98411, O98412, O98413, O98419, O9842, O9843, P7881, Q266, Q446, Q447, R160, R162, R17, R188, R932, R945, T8641, T8642 |
| Heart disease | ICD-10 diagnosis | I201, I208, I209, I25111, I25118, I25119, I25701, I25708, I25709, I25711, I25718, I25719, I25721, I25728, I25729, I25731, I25738, I25739, I25751, I25758, I25759, I25761, I25768, I25769, I25791, I25798, I25799, I200, I25110, I25700, I25710, I25720, I25730, I25750, I25760, I25790, I440, I441, I442, I4430, I4439, I444, I445, I4460, I4469, I447, I450, I4510, I4519, I452, I453, I454, I455, I456, I4581, I4589, I459, I462, I468, I469, I470, I471, I472, I479, I480, I481, I482, I483, I484, I4891, I4892, I4901, I4902, I491, I492, I493, I4940, I4949, I495, I498, I499, R000, R001, R002, R008, R009, Z45010, Z45018, Z4502, Z4509, Z8674, Z950, Z95810, A3282, A3951, A5203, B3321, B376, I011, I330, I339, M3211, I5021, I5031, I5041, I5023, I5033, I5043, I5022, I5032, I5042, I97130, I97131, I0981, I110, I501, I5020, I5030, I5040, I509, I130, I132, I2101, I2102, I2109, I2111, I2119, I2121, I2129, I213, I214, I219, I21A1, I21A9, I220, I221, I222, I228, I229, I230, I231, I232, I233, I234, I235, I236, I238, I510, I511, I512, I237, A3681, A381, A3950, A3952, A3953, A5209, B2682, B3320, B3322, B3323, B3324, B334, B5881, C160, C452, D8685, I010, I012, I018, I019, I020, I029, I090, I092, I0989, I099, I241, I255, I300, I301, I308, I309, I310, I311, I312, I313, I314, I318, I319, I32, I400, I401, I408, I409, I41, I420, I421, I422, I423, I424, I425, I426, I427, I428, I429, I43, I513, I514, I515, I517, I5181, I5189, I519, I52, I970, I97110, I97111, I97120, I97121, I97190, I97191, J1082, J1182, M3212, R010, R011, R012, R0989, R570, R9431, T8111XA, T8111XD, T8111XS, Z95818, Z951, Z955, Z9861, I050, I051, I052, I058, I059, I060, I061, I062, I068, I069, I070, I071, I072, I078, I079, I080, I081, I082, I083, I088, I089, I091, I340, I341, I342, I348, I349, I350, I351, I352, I358, I359, I360, I361, I362, I368, I369, I370, I371, I372, I378, I379, I38, I39, I119, I120, I129, I1310, I1311, I240, I248, I249, I2510, I252, I253, I2541, I2542, I256, I25810, I25811, I25812, I2582, I2583, I2584, I2589, I259, R931, T82211A, T82211D, T82211S, T82212A, T82212D, T82212S, T82213A, T82213D, T82213S, T82218A, T82218D, T82218S |
| Congenital heart defects | ICD-10 diagnosis | Q200, Q201, Q202, Q203, Q204, Q205, Q206, Q208, Q209, Q210, Q211, Q2110, Q2111, Q2112, Q2113, Q2114, Q2115, Q2116, Q2119, Q212, Q2120, Q2121, Q2122, Q2123, Q213, Q214, Q218, Q219, Q220, Q221, Q222, Q223, Q224, Q225, Q226, Q228, Q229, Q230, Q231, Q232, Q233, Q234, Q238, Q239, Q240, Q241, Q242, Q243, Q244, Q245, Q246, Q248, Q249 |
| Stroke | ICD-10 diagnosis | G43601, G43609, G43611, G43619, G460, G461, G462, G463, G464, G465, G466, G467, G468, I6000, I6001, I6002, I6010, I6011, I6012, I602, I6030, I6031, I6032, I604, I6050, I6051, I6052, I606, I607, I608, I609, I610, I611, I612, I613, I614, I615, I616, I618, I619, I6200, I6201, I6202, I6203, I621, I629, I6300, I63011, I63012, I63013, I63019, I6302, I63031, I63032, I63033, I63039, I6309, I6310, I63111, I63112, I63113, I63119, I6312, I63131, I63132, I63133, I63139, I6319, I6320, I63211, I63212, I63213, I63219, I6322, I63231, I63232, I63233, I63239, I6329, I6330, I63311, I63312, I63313, I63319, I63321, I63322, I63323, I63329, I63331, I63332, I63333, I63339, I63341, I63342, I63343, I63349, I6339, I6340, I63411, I63412, I63413, I63419, I63421, I63422, I63423, I63429, I63431, I63432, I63433, I63439, I63441, I63442, I63443, I63449, I6349, I6350, I63511, I63512, I63513, I63519, I63521, I63522, I63523, I63529, I63531, I63532, I63533, I63539, I63541, I63542, I63543, I63549, I6359, I636, I6381, I6389, I639, I6781, I67850, I6900, I69010, I69011, I69012, I69013, I69014, I69015, I69018, I69019, I69020, I69021, I69022, I69023, I69028, I69031, I69032, I69033, I69034, I69039, I69041, I69042, I69043, I69044, I69049, I69051, I69052, I69053, I69054, I69059, I69061, I69062, I69063, I69064, I69065, I69069, I69090, I69091, I69092, I69093, I69098, I6910, I69110, I69111, I69112, I69113, I69114, I69115, I69118, I69119, I69120, I69121, I69122, I69123, I69128, I69131, I69132, I69133, I69134, I69139, I69141, I69142, I69143, I69144, I69149, I69151, I69152, I69153, I69154, I69159, I69161, I69162, I69163, I69164, I69165, I69169, I69190, I69191, I69192, I69193, I69198, I6920, I69210, I69211, I69212, I69213, I69214, I69215, I69218, I69219, I69220, I69221, I69222, I69223, I69228, I69231, I69232, I69233, I69234, I69239, I69241, I69242, I69243, I69244, I69249, I69251, I69252, I69253, I69254, I69259, I69261, I69262, I69263, I69264, I69265, I69269, I69290, I69291, I69292, I69293, I69298, I6930, I69310, I69311, I69312, I69313, I69314, I69315, I69318, I69319, I69320, I69321, I69322, I69323, I69328, I69331, I69332, I69333, I69334, I69339, I69341, I69342, I69343, I69344, I69349, I69351, I69352, I69353, I69354, I69359, I69361, I69362, I69363, I69364, I69365, I69369, I69390, I69391, I69392, I69393, I69398, I97810, I97811, I97820, I97821, S06340A, S06340D, S06340S, S06341A, S06341D, S06341S, S06342A, S06342D, S06342S, S06343A, S06343D, S06343S, S06344A, S06344D, S06344S, S06345A, S06345D, S06345S, S06346A, S06346D, S06346S, S06347A, S06348A, S06349A, S06349D, S06349S, S06350A, S06350D, S06350S, S06351A, S06351D, S06351S, S06352A, S06352D, S06352S, S06353A, S06353D, S06353S, S06354A, S06354D, S06354S, S06355A, S06355D, S06355S, S06356A, S06356D, S06356S, S06357A, S06358A, S06359A, S06359D, S06359S, S06360A, S06360D, S06360S, S06361A, S06361D, S06361S, S06362A, S06362D, S06362S, S06363A, S06363D, S06363S, S06364A, S06364D, S06364S, S06365A, S06365D, S06365S, S06366A, S06366D, S06366S, S06367A, S06368A, S06369A, S06369D, S06369S, S06370A, S06370D, S06370S, S06371A, S06371D, S06371S, S06372A, S06372D, S06372S, S06373A, S06373D, S06373S, S06374A, S06374D, S06374S, S06375A, S06375D, S06375S, S06376A, S06376D, S06376S, S06377A, S06378A, S06379A, S06379D, S06379S, S06380A, S06380D, S06380S, S06381A, S06381D, S06381S, S06382A, S06382D, S06382S, S06383A, S06383D, S06383S, S06384A, S06384D, S06384S, S06385A, S06385D, S06385S, S06386A, S06386D, S06386S, S06387A, S06388A, S06389A, S06389D, S06389S, S064X0A, S064X0D, S064X0S, S064X1A, S064X1D, S064X1S, S064X2A, S064X2D, S064X2S, S064X3A, S064X3D, S064X3S, S064X4A, S064X4D, S064X4S, S064X5A, S064X5D, S064X5S, S064X6A, S064X6D, S064X6S, S064X7A, S064X8A, S064X9A, S064X9D, S064X9S, S065X0A, S065X0D, S065X0S, S065X1A, S065X1D, S065X1S, S065X2A, S065X2D, S065X2S, S065X3A, S065X3D, S065X3S, S065X4A, S065X4D, S065X4S, S065X5A, S065X5D, S065X5S, S065X6A, S065X6D, S065X6S, S065X7A, S065X8A, S065X9A, S065X9D, S065X9S, S066X0A, S066X0D, S066X0S, S066X1A, S066X1D, S066X1S, S066X2A, S066X2D, S066X2S, S066X3A, S066X3D, S066X3S, S066X4A, S066X4D, S066X4S, S066X5A, S066X5D, S066X5S, S066X6A, S066X6D, S066X6S, S066X7A, S066X8A, S066X9A, S066X9D, S066X9S |
| Metabolic disorders | ICD-10 diagnosis | E700, E701, E7020, E7021, E7029, E7030, E70310, E70311, E70318, E70319, E70320, E70321, E70328, E70329, E70330, E70331, E70338, E70339, E7039, E7040, E7041, E7049, E705, E708, E7081, E7089, E709, E710, E71110, E71111, E71118, E71120, E71121, E71128, E7119, E712, E7130, E71310, E71311, E71312, E71313, E71314, E71318, E7132, E7139, E7140, E7141, E7142, E7143, E71440, E71448, E7150, E71510, E71511, E71518, E71520, E71521, E71522, E71528, E71529, E7153, E71540, E71541, E71542, E71548, E7200, E7201, E7202, E7203, E7204, E7209, E7210, E7211, E7212, E7219, E7220, E7221, E7222, E7223, E7229, E723, E724, E7250, E7251, E7252, E7253, E7259, E7281, E7289, E729, E730, E731, E738, E739, E7400, E7401, E7402, E7403, E7404, E7409, E7410, E7411, E7412, E7419, E7420, E7421, E7429, E7431, E7439, E744, E748, E74810, E74818, E74819, E7489, E749, E7500, E7501, E7502, E7509, E7510, E7511, E7519, E7521, E7522, E7523, E75240, E75241, E75242, E75243, E75244, E75248, E75249, E7525, E7526, E7529, E753, E754, E755, E756, E7601, E7602, E7603, E761, E76210, E76211, E76219, E7622, E7629, E763, E768, E769, E770, E771, E778, E779, E7800, E7801, E781, E782, E783, E7841, E7849, E785, E786, E7870, E7871, E7872, E7879, E7881, E7889, E789, E790, E791, E792, E798, E799, E800, E801, E8020, E8021, E8029, E803, E804, E805, E806, E807, E8300, E8301, E8309, E8310, E83110, E83111, E83118, E83119, E8319, E832, E8330, E8331, E8332, E8339, E8340, E8341, E8342, E8349, E8350, E8351, E8352, E8359, E8381, E8389, E839, E840, E8411, E8419, E848, E849, E850, E851, E852, E853, E854, E8581, E8582, E8589, E859, E860, E861, E869, E870, E871, E872, E8720, E8721, E8722, E8729, E873, E874, E875, E876, E8770, E8771, E8779, E878, E8801, E8802, E8809, E881, E882, E883, E8840, E8841, E8842, E8849, E8881, E8889, E889 |
| Current or history of smoking | ICD-10 diagnosis | T65213A, T65213D, T65213S, T65223A, T65223D, T65223S, T65293A, T65293D, T65293S, F17200, F17201, F17203, F17208, F17209, F17210, F17211, F17213, F17218, F17219, F17220, F17221, F17223, F17228, F17229, F17290, F17291, F17293, F17298, F17299, Z87891, T65212A, T65212D, T65212S, T65222A, T65222D, T65222S, T65292A, T65292D, T65292S, Z5731, Z7722, T65211A, T65211D, T65211S, T65221A, T65221D, T65221S, T65291A, T65291D, T65291S, T65214A, T65214D, T65214S, T65224A, T65224D, T65224S, T65294A, T65294D, T65294S, O99330, O99331, O99332, O99333, O99334, O99335, Z716, Z720 |
| Mood disorders | ICD-10 diagnosis | F0630, F0631, F0632, F0633, F0634, F200, F201, F202, F203, F205, F2081, F2089, F209, F21, F250, F251, F258, F259, F3010, F3011, F3012, F3013, F302, F303, F304, F308, F309, F310, F3110, F3111, F3112, F3113, F312, F3130, F3131, F3132, F314, F315, F3160, F3161, F3162, F3163, F3164, F3170, F3171, F3172, F3173, F3174, F3175, F3176, F3177, F3178, F3181, F3189, F319, F320, F321, F322, F323, F324, F325, F328, F3281, F3289, F329, F330, F331, F332, F333, F3340, F3341, F3342, F338, F339, F340, F341, F348, F3481, F3489, F349, F39, F4321, F4323 |
| Blood disorders | ICD-10 diagnosis | D500, D501, D508, D509, D510, D511, D512, D513, D518, D519, D520, D521, D528, D529, D530, D531, D532, D538, D539, D550, D551, D552, D5521, D5529, D553, D558, D559, D560, D561, D562, D563, D564, D565, D568, D569, D5700, D5701, D5702, D5703, D5709, D571, D5720, D57211, D57212, D57213, D57218, D57219, D573, D5740, D57411, D57412, D57413, D57418, D57419, D5742, D57431, D57432, D57433, D57438, D57439, D5744, D57451, D57452, D57453, D57458, D57459, D5780, D57811, D57812, D57813, D57818, D57819, D580, D581, D582, D588, D589, D590, D591, D5910, D5911, D5912, D5913, D5919, D592, D593, D5930, D5931, D5932, D5939, D594, D595, D596, D598, D599, D600, D601, D608, D609, D6101, D6109, D611, D612, D613, D61810, D61811, D61818, D6182, D6189, D619, D62, D630, D631, D638, D640, D641, D642, D643, D644, D6481, D6489, D649, D65, D66, D67, D680, D680, D6800, D6801, D6802, D68020, D68021, D68022, D68023, D68029, D6803, D6804, D6809, D681, D682, D68311, D68312, D68318, D6832, D684, D6851, D6852, D6859, D6861, D6862, D6869, D688, D689, D690, D691, D692, D693, D6941, D6942, D6949, D6951, D6959, D696, D698, D699, D700, D701, D702, D703, D704, D708, D709, D71, D720, D721, D7210, D72110, D72111, D72118, D72119, D7212, D7218, D7219, D72810, D72818, D72819, D72820, D72821, D72822, D72823, D72824, D72825, D72828, D72829, D7289, D729, D730, D731, D732, D733, D734, D735, D7381, D7389, D739, D740, D748, D749, D750, D751, D7581, D7582, D75821, D75822, D75828, D75829, D75838, D75839, D7584, D7589, D759, D75A, D761, D762, D763, D77 |
| Pregnancy | ICD-10 diagnosis | O0000, O0001, O0010, O00101, O00102, O00109, O0011, O00111, O00112, O00119, O0020, O00201, O00202, O00209, O0021, O00211, O00212, O00219, O0080, O0081, O0090, O0091, O010, O011, O019, O020, O021, O0281, O030, O031, O032, O0330, O0331, O0332, O0333, O0334, O0335, O0336, O0337, O0338, O0339, O034, O035, O036, O037, O0380, O0381, O0382, O0383, O0384, O0385, O0386, O0387, O0388, O0389, O039, O045, O046, O047, O0480, O0481, O0482, O0483, O0484, O0485, O0486, O0487, O0488, O0489, O070, O071, O072, O0730, O0731, O0732, O0733, O0734, O0735, O0736, O0737, O0738, O0739, O074, O080, O081, O082, O083, O084, O085, O086, O087, O0881, O0882, O0883, O0889, O089, O0900, O0901, O0902, O0903, O0910, O0911, O0912, O0913, O09211, O09212, O09213, O09219, O09291, O09292, O09293, O09299, O0930, O0931, O0932, O0933, O0940, O0941, O0942, O0943, O09511, O09512, O09513, O09519, O09521, O09522, O09523, O09529, O09611, O09612, O09613, O09619, O09621, O09622, O09623, O09629, O0970, O0971, O0972, O0973, O09811, O09812, O09813, O09819, O09821, O09822, O09823, O09829, O09891, O09892, O09893, O09899, O0990, O0991, O0992, O0993, O09A0, O09A1, O09A2, O09A3, O10011, O10012, O10013, O10019, O1002, O1003, O10111, O10112, O10113, O10119, O1012, O1013, O10211, O10212, O10213, O10219, O1022, O1023, O10311, O10312, O10313, O10319, O1032, O1033, O10411, O10412, O10413, O10419, O1042, O1043, O10911, O10912, O10913, O10919, O1092, O1093, O111, O112, O113, O114, O115, O119, O1200, O1201, O1202, O1203, O1204, O1205, O1210, O1211, O1212, O1213, O1214, O1215, O1220, O1221, O1222, O1223, O1224, O1225, O131, O132, O133, O134, O135, O139, O1400, O1402, O1403, O1404, O1405, O1410, O1412, O1413, O1414, O1415, O1420, O1422, O1423, O1424, O1425, O1490, O1492, O1493, O1494, O1495, O1500, O1502, O1503, O151, O152, O159, O161, O162, O163, O164, O165, O169, O200, O208, O209, O210, O211, O212, O218, O219, O2200, O2201, O2202, O2203, O2210, O2211, O2212, O2213, O2220, O2221, O2222, O2223, O2230, O2231, O2232, O2233, O2240, O2241, O2242, O2243, O2250, O2251, O2252, O2253, O228X1, O228X2, O228X3, O228X9, O2290, O2291, O2292, O2293, O2300, O2301, O2302, O2303, O2310, O2311, O2312, O2313, O2320, O2321, O2322, O2323, O2330, O2331, O2332, O2333, O2340, O2341, O2342, O2343, O23511, O23512, O23513, O23519, O23521, O23522, O23523, O23529, O23591, O23592, O23593, O23599, O2390, O2391, O2392, O2393, O24011, O24012, O24013, O24019, O2402, O2403, O24111, O24112, O24113, O24119, O2412, O2413, O24311, O24312, O24313, O24319, O2432, O2433, O24410, O24414, O24415, O24419, O24420, O24424, O24425, O24429, O24430, O24434, O24435, O24439, O24811, O24812, O24813, O24819, O2482, O2483, O24911, O24912, O24913, O24919, O2492, O2493, O2510, O2511, O2512, O2513, O252, O253, O2600, O2601, O2602, O2603, O2610, O2611, O2612, O2613, O2620, O2621, O2622, O2623, O2630, O2631, O2632, O2633, O2640, O2641, O2642, O2643, O2650, O2651, O2652, O2653, O26611, O26612, O26613, O26619, O2662, O2663, O26711, O26712, O26713, O26719, O2672, O2673, O26811, O26812, O26813, O26819, O26821, O26822, O26823, O26829, O26831, O26832, O26833, O26839, O26841, O26842, O26843, O26849, O26851, O26852, O26853, O26859, O2686, O26872, O26873, O26879, O26891, O26892, O26893, O26899, O2690, O2691, O2692, O2693, O280, O281, O282, O283, O284, O285, O288, O289, O29011, O29012, O29013, O29019, O29021, O29022, O29023, O29029, O29091, O29092, O29093, O29099, O29111, O29112, O29113, O29119, O29121, O29122, O29123, O29129, O29191, O29192, O29193, O29199, O29211, O29212, O29213, O29219, O29291, O29292, O29293, O29299, O293X1, O293X2, O293X3, O293X9, O2940, O2941, O2942, O2943, O295X1, O295X2, O295X3, O295X9, O2960, O2961, O2962, O2963, O298X1, O298X2, O298X3, O298X9, O2990, O2991, O2992, O2993, O30001, O30002, O30003, O30009, O30011, O30012, O30013, O30019, O30021, O30022, O30023, O30029, O30031, O30032, O30033, O30039, O30041, O30042, O30043, O30049, O30091, O30092, O30093, O30099, O30101, O30102, O30103, O30109, O30111, O30112, O30113, O30119, O30121, O30122, O30123, O30129, O30131, O30132, O30133, O30139, O30191, O30192, O30193, O30199, O30201, O30202, O30203, O30209, O30211, O30212, O30213, O30219, O30221, O30222, O30223, O30229, O30231, O30232, O30233, O30239, O30291, O30292, O30293, O30299, O30801, O30802, O30803, O30809, O30811, O30812, O30813, O30819, O30821, O30822, O30823, O30829, O30831, O30832, O30833, O30839, O30891, O30892, O30893, O30899, O3090, O3091, O3092, O3093, O3100X0, O3100X1, O3100X2, O3100X3, O3100X4, O3100X5, O3100X9, O3101X0, O3101X1, O3101X2, O3101X3, O3101X4, O3101X5, O3101X9, O3102X0, O3102X1, O3102X2, O3102X3, O3102X4, O3102X5, O3102X9, O3103X0, O3103X1, O3103X2, O3103X3, O3103X4, O3103X5, O3103X9, O3110X0, O3110X1, O3110X2, O3110X3, O3110X4, O3110X5, O3110X9, O3111X0, O3111X1, O3111X2, O3111X3, O3111X4, O3111X5, O3111X9, O3112X0, O3112X1, O3112X2, O3112X3, O3112X4, O3112X5, O3112X9, O3113X0, O3113X1, O3113X2, O3113X3, O3113X4, O3113X5, O3113X9, O3120X0, O3120X1, O3120X2, O3120X3, O3120X4, O3120X5, O3120X9, O3121X0, O3121X1, O3121X2, O3121X3, O3121X4, O3121X5, O3121X9, O3122X0, O3122X1, O3122X2, O3122X3, O3122X4, O3122X5, O3122X9, O3123X0, O3123X1, O3123X2, O3123X3, O3123X4, O3123X5, O3123X9, O3130X0, O3130X1, O3130X2, O3130X3, O3130X4, O3130X5, O3130X9, O3131X0, O3131X1, O3131X2, O3131X3, O3131X4, O3131X5, O3131X9, O3132X0, O3132X1, O3132X2, O3132X3, O3132X4, O3132X5, O3132X9, O3133X0, O3133X1, O3133X2, O3133X3, O3133X4, O3133X5, O3133X9, O318X10, O318X11, O318X12, O318X13, O318X14, O318X15, O318X19, O318X20, O318X21, O318X22, O318X23, O318X24, O318X25, O318X29, O318X30, O318X31, O318X32, O318X33, O318X34, O318X35, O318X39, O318X90, O318X91, O318X92, O318X93, O318X94, O318X95, O318X99, O320XX0, O320XX1, O320XX2, O320XX3, O320XX4, O320XX5, O320XX9, O321XX0, O321XX1, O321XX2, O321XX3, O321XX4, O321XX5, O321XX9, O322XX0, O322XX1, O322XX2, O322XX3, O322XX4, O322XX5, O322XX9, O323XX0, O323XX1, O323XX2, O323XX3, O323XX4, O323XX5, O323XX9, O324XX0, O324XX1, O324XX2, O324XX3, O324XX4, O324XX5, O324XX9, O326XX0, O326XX1, O326XX2, O326XX3, O326XX4, O326XX5, O326XX9, O328XX0, O328XX1, O328XX2, O328XX3, O328XX4, O328XX5, O328XX9, O329XX0, O329XX1, O329XX2, O329XX3, O329XX4, O329XX5, O329XX9, O330, O331, O332, O333XX0, O333XX1, O333XX2, O333XX3, O333XX4, O333XX5, O333XX9, O334XX0, O334XX1, O334XX2, O334XX3, O334XX4, O334XX5, O334XX9, O335XX0, O335XX1, O335XX2, O335XX3, O335XX4, O335XX5, O335XX9, O336XX0, O336XX1, O336XX2, O336XX3, O336XX4, O336XX5, O336XX9, O337XX0, O337XX1, O337XX2, O337XX3, O337XX4, O337XX5, O337XX9, O338, O339, O3400, O3401, O3402, O3403, O3410, O3411, O3412, O3413, O34211, O34212, O34218, O34219, O3422, O3429, O3430, O3431, O3432, O3433, O3440, O3441, O3442, O3443, O34511, O34512, O34513, O34519, O34521, O34522, O34523, O34529, O34531, O34532, O34533, O34539, O34591, O34592, O34593, O34599, O3460, O3461, O3462, O3463, O3470, O3471, O3472, O3473, O3480, O3481, O3482, O3483, O3490, O3491, O3492, O3493, O3500, O3500X0, O3500X1, O3500X2, O3500X3, O3500X4, O3500X5, O3500X9, O3501, O3501X0, O3501X1, O3501X2, O3501X3, O3501X4, O3501X5, O3501X9, O3502, O3502X0, O3502X1, O3502X2, O3502X3, O3502X4, O3502X5, O3502X9, O3503, O3503X0, O3503X1, O3503X2, O3503X3, O3503X4, O3503X5, O3503X9, O3504, O3504X0, O3504X1, O3504X2, O3504X3, O3504X4, O3504X5, O3504X9, O3505, O3505X0, O3505X1, O3505X2, O3505X3, O3505X4, O3505X5, O3505X9, O3506, O3506X0, O3506X1, O3506X2, O3506X3, O3506X4, O3506X5, O3506X9, O3507, O3507X0, O3507X1, O3507X2, O3507X3, O3507X4, O3507X5, O3507X9, O3508, O3508X0, O3508X1, O3508X2, O3508X3, O3508X4, O3508X5, O3508X9, O3509, O3509X0, O3509X1, O3509X2, O3509X3, O3509X4, O3509X5, O3509X9, O350XX0, O350XX1, O350XX2, O350XX3, O350XX4, O350XX5, O350XX9, O3510, O3510X0, O3510X1, O3510X2, O3510X3, O3510X4, O3510X5, O3510X9, O3511, O3511X0, O3511X1, O3511X2, O3511X3, O3511X4, O3511X5, O3511X9, O3512, O3512X0, O3512X1, O3512X2, O3512X3, O3512X4, O3512X5, O3512X9, O3513, O3513X0, O3513X1, O3513X2, O3513X3, O3513X4, O3513X5, O3513X9, O3514, O3514X0, O3514X1, O3514X2, O3514X3, O3514X4, O3514X5, O3514X9, O3515, O3515X0, O3515X1, O3515X2, O3515X3, O3515X4, O3515X5, O3515X9, O3519, O3519X0, O3519X1, O3519X2, O3519X3, O3519X4, O3519X5, O3519X9, O351XX0, O351XX1, O351XX2, O351XX3, O351XX4, O351XX5, O351XX9, O352XX0, O352XX1, O352XX2, O352XX3, O352XX4, O352XX5, O352XX9, O353XX0, O353XX1, O353XX2, O353XX3, O353XX4, O353XX5, O353XX9, O354XX0, O354XX1, O354XX2, O354XX3, O354XX4, O354XX5, O354XX9, O355XX0, O355XX1, O355XX2, O355XX3, O355XX4, O355XX5, O355XX9, O356XX0, O356XX1, O356XX2, O356XX3, O356XX4, O356XX5, O356XX9, O357XX0, O357XX1, O357XX2, O357XX3, O357XX4, O357XX5, O357XX9, O358XX0, O358XX1, O358XX2, O358XX3, O358XX4, O358XX5, O358XX9, O359XX0, O359XX1, O359XX2, O359XX3, O359XX4, O359XX5, O359XX9, O35A, O35AXX0, O35AXX1, O35AXX2, O35AXX3, O35AXX4, O35AXX5, O35AXX9, O35B, O35BXX0, O35BXX1, O35BXX2, O35BXX3, O35BXX4, O35BXX5, O35BXX9, O35C, O35CXX0, O35CXX1, O35CXX2, O35CXX3, O35CXX4, O35CXX5, O35CXX9, O35D, O35DXX0, O35DXX1, O35DXX2, O35DXX3, O35DXX4, O35DXX5, O35DXX9, O35E, O35EXX0, O35EXX1, O35EXX2, O35EXX3, O35EXX4, O35EXX5, O35EXX9, O35F, O35FXX0, O35FXX1, O35FXX2, O35FXX3, O35FXX4, O35FXX5, O35FXX9, O35G, O35GXX0, O35GXX1, O35GXX2, O35GXX3, O35GXX4, O35GXX5, O35GXX9, O35H, O35HXX0, O35HXX1, O35HXX2, O35HXX3, O35HXX4, O35HXX5, O35HXX9, O360110, O360111, O360112, O360113, O360114, O360115, O360119, O360120, O360121, O360122, O360123, O360124, O360125, O360129, O360130, O360131, O360132, O360133, O360134, O360135, O360139, O360190, O360191, O360192, O360193, O360194, O360195, O360199, O360910, O360911, O360912, O360913, O360914, O360915, O360919, O360920, O360921, O360922, O360923, O360924, O360925, O360929, O360930, O360931, O360932, O360933, O360934, O360935, O360939, O360990, O360991, O360992, O360993, O360994, O360995, O360999, O361110, O361111, O361112, O361113, O361114, O361115, O361119, O361120, O361121, O361122, O361123, O361124, O361125, O361129, O361130, O361131, O361132, O361133, O361134, O361135, O361139, O361190, O361191, O361192, O361193, O361194, O361195, O361199, O361910, O361911, O361912, O361913, O361914, O361915, O361919, O361920, O361921, O361922, O361923, O361924, O361925, O361929, O361930, O361931, O361932, O361933, O361934, O361935, O361939, O361990, O361991, O361992, O361993, O361994, O361995, O361999, O3620X0, O3620X1, O3620X2, O3620X3, O3620X4, O3620X5, O3620X9, O3621X0, O3621X1, O3621X2, O3621X3, O3621X4, O3621X5, O3621X9, O3622X0, O3622X1, O3622X2, O3622X3, O3622X4, O3622X5, O3622X9, O3623X0, O3623X1, O3623X2, O3623X3, O3623X4, O3623X5, O3623X9, O364XX0, O364XX1, O364XX2, O364XX3, O364XX4, O364XX5, O364XX9, O365110, O365111, O365112, O365113, O365114, O365115, O365119, O365120, O365121, O365122, O365123, O365124, O365125, O365129, O365130, O365131, O365132, O365133, O365134, O365135, O365139, O365190, O365191, O365192, O365193, O365194, O365195, O365199, O365910, O365911, O365912, O365913, O365914, O365915, O365919, O365920, O365921, O365922, O365923, O365924, O365925, O365929, O365930, O365931, O365932, O365933, O365934, O365935, O365939, O365990, O365991, O365992, O365993, O365994, O365995, O365999, O3660X0, O3660X1, O3660X2, O3660X3, O3660X4, O3660X5, O3660X9, O3661X0, O3661X1, O3661X2, O3661X3, O3661X4, O3661X5, O3661X9, O3662X0, O3662X1, O3662X2, O3662X3, O3662X4, O3662X5, O3662X9, O3663X0, O3663X1, O3663X2, O3663X3, O3663X4, O3663X5, O3663X9, O3670X0, O3670X1, O3670X2, O3670X3, O3670X4, O3670X5, O3670X9, O3671X0, O3671X1, O3671X2, O3671X3, O3671X4, O3671X5, O3671X9, O3672X0, O3672X1, O3672X2, O3672X3, O3672X4, O3672X5, O3672X9, O3673X0, O3673X1, O3673X2, O3673X3, O3673X4, O3673X5, O3673X9, O3680X0, O3680X1, O3680X2, O3680X3, O3680X4, O3680X5, O3680X9, O368120, O368121, O368122, O368123, O368124, O368125, O368129, O368130, O368131, O368132, O368133, O368134, O368135, O368139, O368190, O368191, O368192, O368193, O368194, O368195, O368199, O368210, O368211, O368212, O368213, O368214, O368215, O368219, O368220, O368221, O368222, O368223, O368224, O368225, O368229, O368230, O368231, O368232, O368233, O368234, O368235, O368239, O368290, O368291, O368292, O368293, O368294, O368295, O368299, O368310, O368311, O368312, O368313, O368314, O368315, O368319, O368320, O368321, O368322, O368323, O368324, O368325, O368329, O368330, O368331, O368332, O368333, O368334, O368335, O368339, O368390, O368391, O368392, O368393, O368394, O368395, O368399, O368910, O368911, O368912, O368913, O368914, O368915, O368919, O368920, O368921, O368922, O368923, O368924, O368925, O368929, O368930, O368931, O368932, O368933, O368934, O368935, O368939, O368990, O368991, O368992, O368993, O368994, O368995, O368999, O3690X0, O3690X1, O3690X2, O3690X3, O3690X4, O3690X5, O3690X9, O3691X0, O3691X1, O3691X2, O3691X3, O3691X4, O3691X5, O3691X9, O3692X0, O3692X1, O3692X2, O3692X3, O3692X4, O3692X5, O3692X9, O3693X0, O3693X1, O3693X2, O3693X3, O3693X4, O3693X5, O3693X9, O401XX0, O401XX1, O401XX2, O401XX3, O401XX4, O401XX5, O401XX9, O402XX0, O402XX1, O402XX2, O402XX3, O402XX4, O402XX5, O402XX9, O403XX0, O403XX1, O403XX2, O403XX3, O403XX4, O403XX5, O403XX9, O409XX0, O409XX1, O409XX2, O409XX3, O409XX4, O409XX5, O409XX9, O4100X0, O4100X1, O4100X2, O4100X3, O4100X4, O4100X5, O4100X9, O4101X0, O4101X1, O4101X2, O4101X3, O4101X4, O4101X5, O4101X9, O4102X0, O4102X1, O4102X2, O4102X3, O4102X4, O4102X5, O4102X9, O4103X0, O4103X1, O4103X2, O4103X3, O4103X4, O4103X5, O4103X9, O411010, O411011, O411012, O411013, O411014, O411015, O411019, O411020, O411021, O411022, O411023, O411024, O411025, O411029, O411030, O411031, O411032, O411033, O411034, O411035, O411039, O411090, O411091, O411092, O411093, O411094, O411095, O411099, O411210, O411211, O411212, O411213, O411214, O411215, O411219, O411220, O411221, O411222, O411223, O411224, O411225, O411229, O411230, O411231, O411232, O411233, O411234, O411235, O411239, O411290, O411291, O411292, O411293, O411294, O411295, O411299, O411410, O411411, O411412, O411413, O411414, O411415, O411419, O411420, O411421, O411422, O411423, O411424, O411425, O411429, O411430, O411431, O411432, O411433, O411434, O411435, O411439, O411490, O411491, O411492, O411493, O411494, O411495, O411499, O418X10, O418X11, O418X12, O418X13, O418X14, O418X15, O418X19, O418X20, O418X21, O418X22, O418X23, O418X24, O418X25, O418X29, O418X30, O418X31, O418X32, O418X33, O418X34, O418X35, O418X39, O418X90, O418X91, O418X92, O418X93, O418X94, O418X95, O418X99, O4190X0, O4190X1, O4190X2, O4190X3, O4190X4, O4190X5, O4190X9, O4191X0, O4191X1, O4191X2, O4191X3, O4191X4, O4191X5, O4191X9, O4192X0, O4192X1, O4192X2, O4192X3, O4192X4, O4192X5, O4192X9, O4193X0, O4193X1, O4193X2, O4193X3, O4193X4, O4193X5, O4193X9, O4200, O42011, O42012, O42013, O42019, O4202, O4210, O42111, O42112, O42113, O42119, O4212, O4290, O42911, O42912, O42913, O42919, O4292, O43011, O43012, O43013, O43019, O43021, O43022, O43023, O43029, O43101, O43102, O43103, O43109, O43111, O43112, O43113, O43119, O43121, O43122, O43123, O43129, O43191, O43192, O43193, O43199, O43211, O43212, O43213, O43219, O43221, O43222, O43223, O43229, O43231, O43232, O43233, O43239, O43811, O43812, O43813, O43819, O43891, O43892, O43893, O43899, O4390, O4391, O4392, O4393, O4400, O4401, O4402, O4403, O4410, O4411, O4412, O4413, O4420, O4421, O4422, O4423, O4430, O4431, O4432, O4433, O4440, O4441, O4442, O4443, O4450, O4451, O4452, O4453, O45001, O45002, O45003, O45009, O45011, O45012, O45013, O45019, O45021, O45022, O45023, O45029, O45091, O45092, O45093, O45099, O458X1, O458X2, O458X3, O458X9, O4590, O4591, O4592, O4593, O46001, O46002, O46003, O46009, O46011, O46012, O46013, O46019, O46021, O46022, O46023, O46029, O46091, O46092, O46093, O46099, O468X1, O468X2, O468X3, O468X9, O4690, O4691, O4692, O4693, O4700, O4702, O4703, O471, O479, O480, O481, O6000, O6002, O6003, O6010X0, O6010X1, O6010X2, O6010X3, O6010X4, O6010X5, O6010X9, O6012X0, O6012X1, O6012X2, O6012X3, O6012X4, O6012X5, O6012X9, O6013X0, O6013X1, O6013X2, O6013X3, O6013X4, O6013X5, O6013X9, O6014X0, O6014X1, O6014X2, O6014X3, O6014X4, O6014X5, O6014X9, O6020X0, O6020X1, O6020X2, O6020X3, O6020X4, O6020X5, O6020X9, O6022X0, O6022X1, O6022X2, O6022X3, O6022X4, O6022X5, O6022X9, O6023X0, O6023X1, O6023X2, O6023X3, O6023X4, O6023X5, O6023X9, O610, O611, O618, O619, O620, O621, O622, O623, O624, O628, O629, O630, O631, O632, O639, O640XX0, O640XX1, O640XX2, O640XX3, O640XX4, O640XX5, O640XX9, O641XX0, O641XX1, O641XX2, O641XX3, O641XX4, O641XX5, O641XX9, O642XX0, O642XX1, O642XX2, O642XX3, O642XX4, O642XX5, O642XX9, O643XX0, O643XX1, O643XX2, O643XX3, O643XX4, O643XX5, O643XX9, O644XX0, O644XX1, O644XX2, O644XX3, O644XX4, O644XX5, O644XX9, O645XX0, O645XX1, O645XX2, O645XX3, O645XX4, O645XX5, O645XX9, O648XX0, O648XX1, O648XX2, O648XX3, O648XX4, O648XX5, O648XX9, O649XX0, O649XX1, O649XX2, O649XX3, O649XX4, O649XX5, O649XX9, O650, O651, O652, O653, O654, O655, O658, O659, O660, O661, O662, O663, O6640, O6641, O665, O666, O668, O669, O670, O678, O679, O68, O690XX0, O690XX1, O690XX2, O690XX3, O690XX4, O690XX5, O690XX9, O691XX0, O691XX1, O691XX2, O691XX3, O691XX4, O691XX5, O691XX9, O692XX0, O692XX1, O692XX2, O692XX3, O692XX4, O692XX5, O692XX9, O693XX0, O693XX1, O693XX2, O693XX3, O693XX4, O693XX5, O693XX9, O694XX0, O694XX1, O694XX2, O694XX3, O694XX4, O694XX5, O694XX9, O695XX0, O695XX1, O695XX2, O695XX3, O695XX4, O695XX5, O695XX9, O6981X0, O6981X1, O6981X2, O6981X3, O6981X4, O6981X5, O6981X9, O6982X0, O6982X1, O6982X2, O6982X3, O6982X4, O6982X5, O6982X9, O6989X0, O6989X1, O6989X2, O6989X3, O6989X4, O6989X5, O6989X9, O699XX0, O699XX1, O699XX2, O699XX3, O699XX4, O699XX5, O699XX9, O700, O701, O7020, O7021, O7022, O7023, O703, O704, O709, O7100, O7102, O7103, O711, O712, O713, O714, O715, O716, O717, O7181, O7182, O7189, O719, O720, O721, O722, O723, O730, O731, O740, O741, O742, O743, O744, O745, O746, O747, O748, O749, O750, O751, O752, O753, O754, O755, O7581, O7582, O7589, O759, O76, O770, O771, O778, O779, O80, O82, O85, O8600, O8601, O8602, O8603, O8604, O8609, O8611, O8612, O8613, O8619, O8620, O8621, O8622, O8629, O864, O8681, O8689, O870, O871, O872, O873, O874, O878, O879, O88011, O88012, O88013, O88019, O8802, O8803, O88111, O88112, O88113, O88119, O8812, O8813, O88211, O88212, O88213, O88219, O8822, O8823, O88311, O88312, O88313, O88319, O8832, O8833, O88811, O88812, O88813, O88819, O8882, O8883, O8901, O8909, O891, O892, O893, O894, O895, O896, O898, O899, O900, O901, O902, O903, O904, O905, O906, O9081, O9089, O909, O91011, O91012, O91013, O91019, O9102, O9103, O91111, O91112, O91113, O91119, O9112, O9113, O91211, O91212, O91213, O91219, O9122, O9123, O92011, O92012, O92013, O92019, O9202, O9203, O92111, O92112, O92113, O92119, O9212, O9213, O9220, O9229, O923, O924, O925, O926, O9270, O9279, O94, O98011, O98012, O98013, O98019, O9802, O9803, O98111, O98112, O98113, O98119, O9812, O9813, O98211, O98212, O98213, O98219, O9822, O9823, O98311, O98312, O98313, O98319, O9832, O9833, O98411, O98412, O98413, O98419, O9842, O9843, O98511, O98512, O98513, O98519, O9852, O9853, O98611, O98612, O98613, O98619, O9862, O9863, O98711, O98712, O98713, O98719, O9872, O9873, O98811, O98812, O98813, O98819, O9882, O9883, O98911, O98912, O98913, O98919, O9892, O9893, O99011, O99012, O99013, O99019, O9902, O9903, O99111, O99112, O99113, O99119, O9912, O9913, O99210, O99211, O99212, O99213, O99214, O99215, O99280, O99281, O99282, O99283, O99284, O99285, O99310, O99311, O99312, O99313, O99314, O99315, O99320, O99321, O99322, O99323, O99324, O99325, O99330, O99331, O99332, O99333, O99334, O99335, O99340, O99341, O99342, O99343, O99344, O99345, O99350, O99351, O99352, O99353, O99354, O99355, O99411, O99412, O99413, O99419, O9942, O9943, O99511, O99512, O99513, O99519, O9952, O9953, O99611, O99612, O99613, O99619, O9962, O9963, O99711, O99712, O99713, O99719, O9972, O9973, O99810, O99814, O99815, O99820, O99824, O99825, O99830, O99834, O99835, O99840, O99841, O99842, O99843, O99844, O99845, O9989, O99891, O99892, O99893, O9A111, O9A112, O9A113, O9A119, O9A12, O9A13, O9A211, O9A212, O9A213, O9A219, O9A22, O9A23, O9A311, O9A312, O9A313, O9A319, O9A32, O9A33, O9A411, O9A412, O9A413, O9A419, O9A42, O9A43, O9A511, O9A512, O9A513, O9A519, O9A52, O9A53, Z1332, Z3201, Z331, Z332, Z333, Z3400, Z3401, Z3402, Z3403, Z3480, Z3481, Z3482, Z3483, Z3490, Z3491, Z3492, Z3493, Z36, Z360, Z361, Z362, Z363, Z365, Z3682, Z3685, Z3686, Z3687, Z3689, Z368A, Z369, Z370, Z371, Z372, Z373, Z374, Z3750, Z3751, Z3752, Z3753, Z3754, Z3759, Z3760, Z3761, Z3762, Z3763, Z3764, Z3769, Z377, Z379, Z390, Z391, Z392, Z3A00, Z3A01, Z3A08, Z3A09, Z3A10, Z3A11, Z3A12, Z3A13, Z3A14, Z3A15, Z3A16, Z3A17, Z3A18, Z3A19, Z3A20, Z3A21, Z3A22, Z3A23, Z3A24, Z3A25, Z3A26, Z3A27, Z3A28, Z3A29, Z3A30, Z3A31, Z3A32, Z3A33, Z3A34, Z3A35, Z3A36, Z3A37, Z3A38, Z3A39, Z3A40, Z3A41, Z3A42, Z3A49, Z640 |
|  | CPT | 36460, 57022, 58605, 58974, 58976, 59000, 59001, 59012, 59015, 59020, 59025, 59030, 59050, 59051, 59070, 59072, 59074, 59076, 59100, 59120, 59121, 59130, 59135, 59136, 59140, 59150, 59151, 59160, 59200, 59300, 59320, 59325, 59350, 59400, 59409, 59410, 59412, 59414, 59425, 59426, 59430, 59510, 59514, 59515, 59525, 59610, 59612, 59614, 59618, 59620, 59622, 59812, 59820, 59821, 59830, 59840, 59841, 59850, 59851, 59852, 59855, 59856, 59857, 59866, 59870, 59871, 59897, 59898, 59899, 74712, 74713, 76801, 76802, 76805, 76810, 76811, 76812, 76813, 76814, 76815, 76816, 76817, 76818, 76819, 76820, 76821, 76825, 76826, 76827, 76828, 76941, 76945, 76946, 80055, 80081, 81422, 81507, 81508, 81509, 81510, 81511, 81512, 82106, 82731, 83030, 83033, 83661, 83662, 83663, 83664, 85460, 85461, 99500, 0060U, 0168U, 01960, 01961, 01962, 01963, 01965, 01966, 01967, 01968, 01969, 0252U, 0341U, 0475T, 0476T, 0477T, 0478T, 81420 |
|  | HCPCS | H1000, H1001, H1002, H1003, H1004, H1005, S0197, S0199, S2260, S2265, S2266, S2267, S2400, S2401, S2402, S2403, S2404, S2405, S2409, S2411, S8055, S9212, S9436, S9437, S9438, S9439, S9442, S9443 |
| Obesity | ICD-10 diagnosis | Z6830, Z6831, Z6832, Z6833, Z6834, Z6835, Z6836, Z6837, Z6838, Z6839, Z6841, Z6842, Z6843, Z6844, Z6845, Z6854, E661, E6601, E662, O99214, O99211, O99212, O99213, O99210, O99215, E669, E668, E6609 |
|  | HCPCS | G0447, G0473 |

CPT = Current Procedural Terminology, HCPCS = Healthcare Common Procedure Coding System, ICD-10 = International Classification of Diseases 10th Revision
