# Supplementary material for: Real-world use of multiplex point-of-care molecular testing or laboratory-based molecular testing for influenza-like illness in a 2021 to 2022 US outpatient sample
Source: PLoS One. 2024 Nov 11;19(11):e0313660. doi: 10.1371/journal.pone.0313660 (PMC11554232; doi:10.1371/journal.pone.0313660)
Supplement: S6 Table — (DOCX) [file pone.0313660.s006.docx]

# S6 Table. Diagnosis Codes for Active COVID-19, Influenza, and RSV

| **ICD-10 Diagnosis Code** | **Description** | **Active COVID-19** | **Active Influenza** | **Active RSV** |
| --- | --- | --- | --- | --- |
| J1282 | Pneumonia due to coronavirus disease 2019 | X |  |  |
| U071 | COVID-19 | X |  |  |
| J09X1 | Influenza due to identified novel influenza A virus with pneumonia |  | X |  |
| J09X2 | Influenza due to identified novel influenza A virus with other respiratory manifestations |  | X |  |
| J09X3 | Influenza due to identified novel influenza A virus with gastrointestinal manifestations |  | X |  |
| J09X9 | Influenza due to identified novel influenza A virus with other manifestations |  | X |  |
| J1000 | Influenza due to other identified influenza virus with unspecified type of pneumonia |  | X |  |
| J1001 | Influenza due to other identified influenza virus with the same other identified influenza virus pneumonia |  | X |  |
| J1008 | Influenza due to other identified influenza virus with other specified pneumonia |  | X |  |
| J101 | Influenza due to other identified influenza virus with other respiratory manifestations |  | X |  |
| J102 | Influenza due to other identified influenza virus with gastrointestinal manifestations |  | X |  |
| J1081 | Influenza due to other identified influenza virus with encephalopathy |  | X |  |
| J1082 | Influenza due to other identified influenza virus with myocarditis |  | X |  |
| J1083 | Influenza due to other identified influenza virus with otitis media |  | X |  |
| J1089 | Influenza due to other identified influenza virus with other manifestations |  | X |  |
| J1100 | Influenza due to unidentified influenza virus with unspecified type of pneumonia |  | X |  |
| J1108 | Influenza due to unidentified influenza virus with specified pneumonia |  | X |  |
| J111 | Influenza due to unidentified influenza virus with other respiratory manifestations |  | X |  |
| J112 | Influenza due to unidentified influenza virus with gastrointestinal manifestations |  | X |  |
| J1181 | Influenza due to unidentified influenza virus with encephalopathy |  | X |  |
| J1182 | Influenza due to unidentified influenza virus with myocarditis |  | X |  |
| J1183 | Influenza due to unidentified influenza virus with otitis media |  | X |  |
| J1189 | Influenza due to unidentified influenza virus with other manifestations |  | X |  |
| B974 | Respiratory syncytial virus as the cause of diseases classified elsewhere |  |  | X |
| J121 | Respiratory syncytial virus pneumonia |  |  | X |
| J205 | Acute bronchitis due to respiratory syncytial virus |  |  | X |
| J210 | Acute bronchiolitis due to respiratory syncytial virus |  |  | X |

ICD-10 = International Classification of Diseases 10th Revision, COVID-19 = coronavirus disease 2019, RSV = respiratory syncytial virus
