# Supplementary material for: Real-world use of multiplex point-of-care molecular testing or laboratory-based molecular testing for influenza-like illness in a 2021 to 2022 US outpatient sample
Source: PLoS One. 2024 Nov 11;19(11):e0313660. doi: 10.1371/journal.pone.0313660 (PMC11554232; doi:10.1371/journal.pone.0313660)
Supplement: S7 Table — (DOCX) [file pone.0313660.s007.docx]

# S7 Table. Treatments for COVID-19, Influenza, and RSV

| **Condition** | **Medication Category** | **Generic Name** |
| --- | --- | --- |
| COVID-19 | Antivirals | ritonavir-boosted nirmatrelvir, remdesivir, molnupiravir |
|  | Immunomodulators and JAK inhibitors | baricitinib, tofacitinib, tocalizumab, sarilumab |
|  | Monoclonal antibodies | bamlanivimab, etesevimab, casirivimab, imdevimab, sotrovimab, bebtelovimab, tixagevimab,  cilgavimab |
|  | Other treatments | hydroxychloroquine, convalescent plasma |
| Influenza | Antivirals | baloxavir, oseltamivir, peramivir, rimatadine, zanamivir |
| RSV | Antivirals | ribavirin |
|  | Monoclonal antibodies | palivizumab |
|  | Immune serums | immune globulins |

COVID-19 = coronavirus disease 2019, JAK = Janus kinase inhibitors, RSV = respiratory syncytial virus
